# Analysis of Micro-Rearrangements in 25 Eukaryotic Species Pairs by SyntenyMapper

**DOI:** 10.1371/journal.pone.0112341

**Published:** 2014-11-06

**Authors:** Stefanie Kaufmann, Dmitrij Frishman

**Affiliations:** 1 Department of Genome Oriented Bioinformatics, Technische Universität München, Freising, Bavaria, Germany; 2 Institute of Bioinformatics and Systems Biology, German Research Center for Environmental Health, Neuherberg, Bavaria, Germany; 3 Department of Bioinformatics, St Petersburg State Polytechnical University, St Petersburg, Russia; Macquarie University, Australia

## Abstract

High-quality mapping of genomic regions and genes between two organisms is an indispensable prerequisite for evolutionary analyses and comparative genomics. Existing approaches to this problem focus on either delineating orthologs or finding extended sequence regions of common evolutionary origin (syntenic blocks). We propose SyntenyMapper, a novel tool for refining predefined syntenic regions. SyntenyMapper creates a set of blocks with conserved gene order between two genomes and finds all minor rearrangements that occurred since the evolutionary split of the two species considered. We also present TrackMapper, a SyntenyMapper-based tool that allows users to directly compare genome features, such as histone modifications, between two organisms, and identify genes with highly conserved features. We demonstrate SyntenyMapper's advantages by conducting a large-scale analysis of micro-rearrangements within syntenic regions of 25 eukaryotic species. Unsurprisingly, the number and length of syntenic regions is correlated with evolutionary distance, while the number of micro-rearrangements depends only on the size of the harboring region. On the other hand, the size of rearranged regions remains relatively constant regardless of the evolutionary distance between the organisms, implying a length constraint in the rearrangement process. SyntenyMapper is a useful software tool for both large-scale and gene-centric genome comparisons.

## Introduction

The most basic step in comparative genomics is to find functional genetic elements (genes, pseudogenes, repeats, regulatory sequences) as well as entire genome regions that are conserved between two species. The finding of matching genomic regions is central to tracing the evolutionary history that led from a common ancestor to the contemporary genome sequences via a succession of evolutionary events, such as gene duplications and translocations. Establishing equivalent locations between genomes is also an important prerequisite for comparing position-specific functional, structural, and evolutionary features measured by modern high-throughput techniques, such as transcriptionally active regions, chromatin accessibility, replication domains and single nucleotide polymorphisms (SNPs).

Finding corresponding locations in two different genomes usually involves the identification of syntenic regions, which represent the longest sequence stretches of common evolutionary origin and consist of a number of conserved genome regions, often with interspersed short segments of lower or no similarity [1]. The order of these equivalent syntenic regions is different in both genomes due to an unknown number of large rearrangement events that occurred after the species diverged from the last common ancestor. Based on their extent, rearrangements are usually (somewhat arbitrarily) subdivided into two classes: i) macro-rearrangements, which involve multi-megabase sized intra- and interchromosomal relocation of large syntenic blocks, and ii) micro-rearrangements [1], i.e. re-ordering of smaller segments (below 1Mb) within a syntenic region.

**Figure 1:**
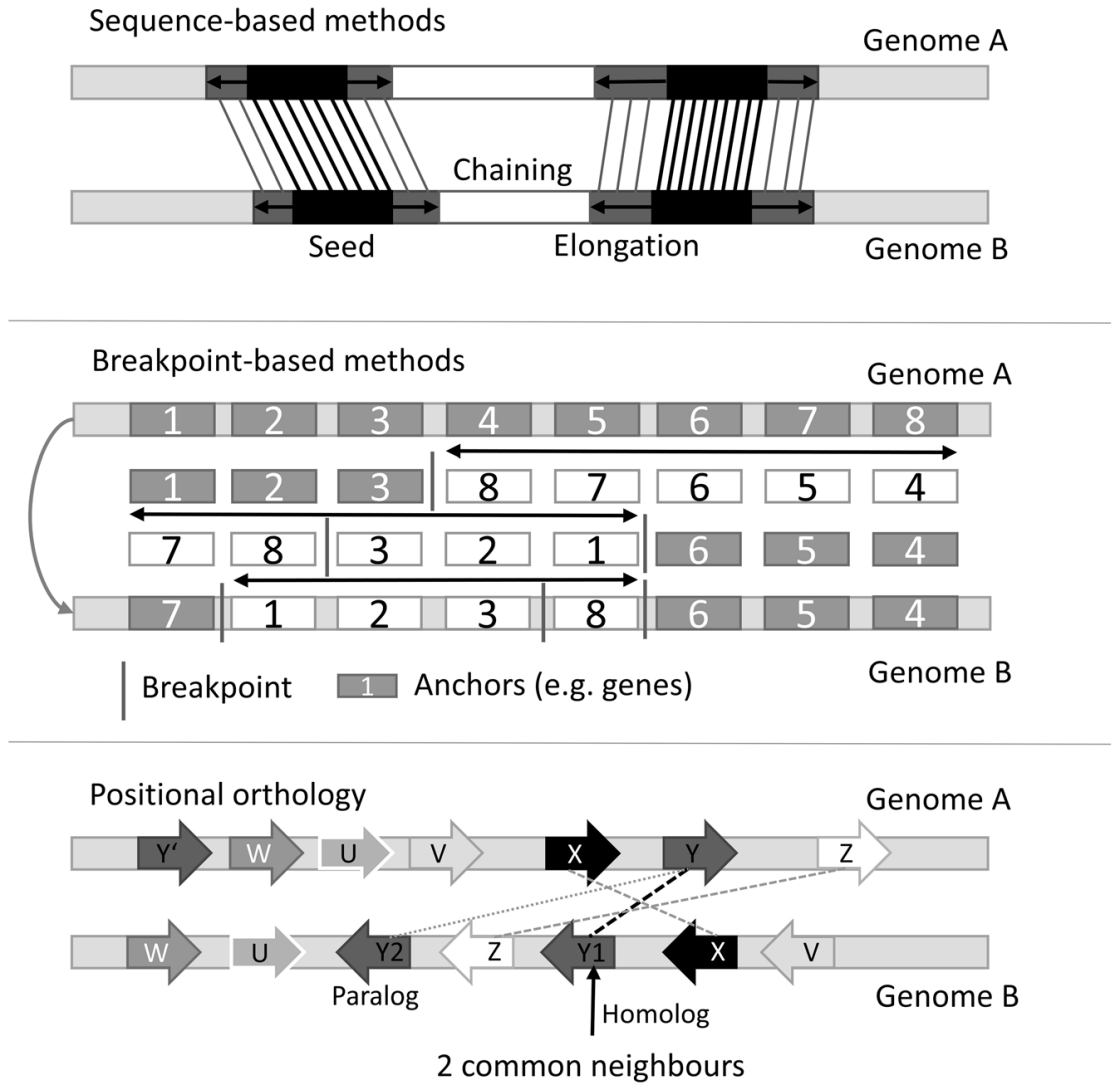
Overview of different approaches for identifying orthologous regions in two genomes. Sequence-based methods (e.g. ENSEMBL Compara) start with short local alignments that are extended to longest possible alignments over gaps. Breakpoint-based methods use orthologous elements (called ‘anchors') to find the minimum number of rearrangements that transforms one genome into the other. Positional orthology tries to distinguish orthologs from paralogs by analyzing gene neighborhoods.

The distinction between finding large regions of common origin and rearrangements of gene order at a much finer scale is intrinsically ill defined. In addition, each of these tasks has its own complications. While looking for long genomic blocks in two genomes that have evolved from the same sequence, one must allow for gaps and ignore micro-rearrangements. The search for orthologs is hampered by the presence of paralogous families and by local similarity hits covering individual domains of multi-domain proteins. Below we give a general overview of the most common types of methods for the identification of equivalent genomic locations using synteny, as illustrated in Figure 1.

### Sequence-based methods

The most widely used approach to syntenic block detection is based on whole-genome sequence alignments. For example, ENSEMBL Compara [2], [3] starts with short local alignment seeds with a perfect match in both organisms and elongates them until the similarity score falls below a predefined threshold. This procedure produces a set of medium length alignments with a low number of gaps and mismatches. Pairs of such alignments that are located sufficiently close to each other are then chained to create extended aligned stretches of common evolutionary origin. A similar approach was published by Liao et al. [4] who introduced pairs of unique 16-mers as a substitute for local alignments. This allows for a much faster searching for orthologous regions than whole-genome alignments. The method is designed to find the longest regions with a common ancestor in two genomes and is thus appropriate for analyzing large-scale genomic rearrangement events, but not for comparisons at the gene level.

### Breakpoint-based methods

These methods [1] divide both genomes to be compared into homologous elements, e.g. genes that can be matched between them. The goal is to find the smallest combination of translocations, inversions and duplications explaining today's genomes. One example of a breakpoint-based method for the detection of syntenic blocks is the GRIMM genome rearrangements Web server [5], which is based on the popular algorithm by Hannenhalli and Pevzner [6], [7]. GRIMM is able to distinguish between macro- and micro-rearrangements and relies on an initial alignment of orthologous elements. However, since GRIMM's main goal is the reconstruction of evolutionary events, it focuses on finding macro-rearrangements and the most important micro-rearrangements within them while discarding small rearrangements. While it is a well-suited method for the detection of macro-rearrangements, it fails to reconstruct a complete evolutionary history on the level of genes. Additionally, GRIMM is not able to deal with gene duplication in a genome (the so called ‘word problem’). Another method, also based on the Hannenhalli-Pevzner algorithm, is Cinteny [8], which focuses on the detection of syntenic regions at different sensitivities.

### Positional orthology

Positional orthology is an umbrella term for all ortholog prediction methods that rely on gene neighborhood, such as localSynteny [9] or MSOAR [10], [11]. They aim to produce a one-to-one mapping of genes by taking the direct neighbors of all orthologs into account and attempt to delineate orthologs based on the evolutionary time point of their creation. This leads to a mapping with higher probabilities for the resulting orthologs to fulfill similar functions in their species. The main drawback of these methods is their definition of gene neighborhood, which is composed of the adjacent genes on both sides. Only a subset of direct neighbors is thus used to define the orthology of the genes in question, although consideration of a larger segment of genes, which lies in a conserved order in both genomes could greatly improve the prediction.

All types of approaches focus on one partial aspect of the evolutionary relationship between two species, even though they are highly interdependent, and no single approach, applied separately, is adequate for comparing quantitative or qualitative properties along eukaryotic chromosomes. Comparative genomics studies focusing on genes and their immediate environment (i.e. intergenic regions, regulatory elements, adjacent genes) usually only compare orthologs. If the immediate linear environment of the gene is important for the comparison of species, considering pairs of orthologs independently from each other is not sufficient. For example, the expression of Homeobox (Hox) genes is determined by their order on the chromosome [12], [13]. Another example are lamina- associated domains (LAD) which, when located in the intergenic region between two genes, determine the subnuclear localization of the entire genomic region. Features such as LADs are known to correlate with hetero- and euchromatin [14] and hence also influence gene expression, which, again, demonstrates the importance of the linear environment in comparative genomics.

Syntenic regions, originally defined as segments of conserved gene order, would seem to be the perfect basis for comparing both genes and their neighborhoods between two species, but there are very few regions of continuous similarity between mammalian genomes. Instead, a more general term syntenic blocks is often used [1] to describe conserved regions that are interrupted by local micro-rearrangements and therefore do not lend themselves to straightforward comparison of genes and their environments. Refinement of such regions formed by macro-rearrangements and delineation of maximal length blocks of conserved gene order are required to allow for comparison of genes and regulatory elements in conserved neighborhoods.

In this work we propose a new method, SyntenyMapper, which aims to reconcile all three types of approaches discussed above by combining the search for completely conserved blocks of orthologous genes with the identification of microrearrangements within syntenic blocks. Using pre-calculated syntenic regions and orthologous genes as input, SyntenyMapper finds rearranged regions of conserved gene order within syntenic blocks. It is thus well suited for a gene-based comparison of genomes, since it not only allows for consideration of the genomic properties of orthologous genes, but also of their conserved gene neighborhood.

SyntenyMapper can be best compared with a class of orthology-based tools suitable for the identification of small blocks of conserved gene order, or collinear blocks, which usually work on a genome-wide scale. Similar to our tool, CYNTENATOR [15], MCScanX [16] and i-ADHoRe [17] identify regions of conserved gene order by applying alignment techniques to a set of ortholog anchors such as genes. These methods are in principle suitable for identifying all micro-rearrangements of gene order in a genome and are comparable to SyntenyMapper when applied to syntenic regions (see the Results section for a detailed comparison). However, in contrast to these methods, SyntenyMapper is designed to take into account the hierarchical structure of the genome and aims to find *all* exact micro-rearrangements within predefined syntenic blocks, independent of the number of elements they contain. CYNTENATOR, i-ADHoRe and MCScanX are less precise, allowing for gaps and mismatches of ortholog pairs. Our tool, however, delineates small blocks of perfectly conserved order that are ideal for comparison of closely related genomes and analyzing evolutionary rearrangement events in a region of interest. Through the use of predefined syntenic regions, the complexity of the task is greatly reduced and SyntenyMapper is thus able to find all small rearrangements within seconds.

Additionally, SyntenyMapper implements a preprocessing step to create a set of syntenic one-to-one orthologs. It thus also has a lot in common with positional orthology methods. However, SyntenyMapper relies on known orthology relationships and filters many-to-many groups according to gene order on the chromosome. The conservation of gene order in one large and conserved segment is thus the main guide for creating a one-to-one orthology mapping. SyntenyMapper expands and combines existing approaches for a more detailed assessment of evolutionary history.

SyntenyMapper is available as a stand-alone software repository via the Galaxy [18], [19], [20] platform's tool shed (https://toolshed.g2.bx.psu.edu, repository name “synteny_mapper”) and as source code on our website (http://webclu.bio.wzw.tum.de/syntenymapper). Pre-computed results for the syntenic regions and orthologs from the ENSEMBL Compara database [2], [3] can be accessed and downloaded at our website as well.

## Material and Methods

### SyntenyMapper

**Figure 2:**
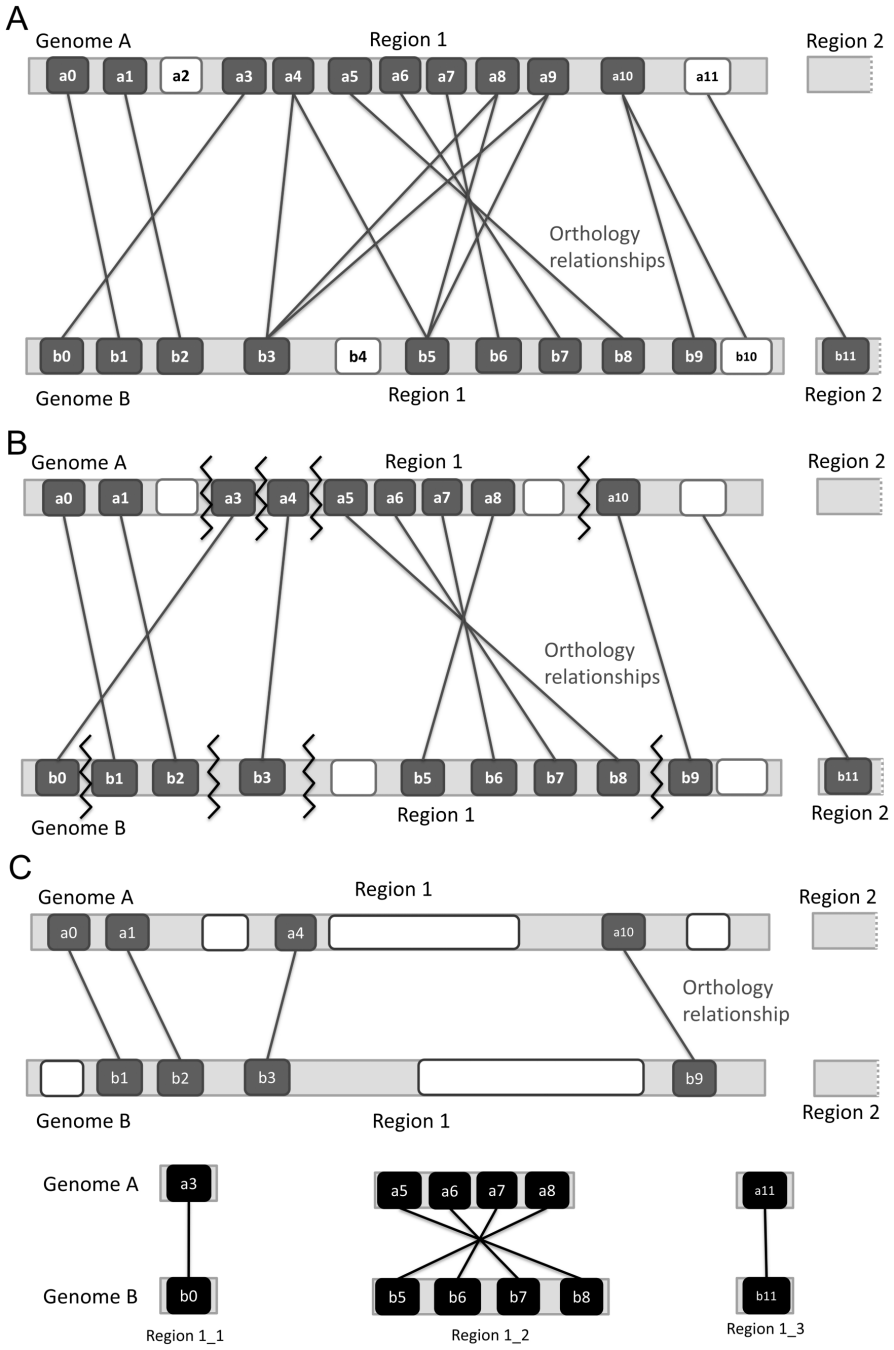
Detection of collinear blocks by SyntenyMapper. A: Illustration of a syntenic region between two species, with numbered boxes representing genes and connecting lines representing orthology relationships. Gene 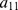 and gene 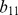 have no orthologs in their syntenic regions, but are orthologous to each other. Genes 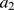 and 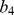 have no orthologs. B: During pre-processing one-to-many (genes 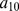 and 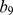, 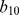) and asymmetric many-to-many (genes 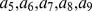 and 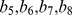), groups are first converted into symmetric groups by excluding genes with the lowest sequence identity to the rest of the group (genes 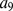 and 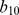), and subsequently paired as one-to-one orthologs based on gene order. Breakpoints (zig-zag lines) are identified as described in the Methods section. C: Using breakpoints, SyntenyMapper defines rearranged segments, shown in black, as new syntenic regions 1_1 to 1_3 within the long original region.

SyntenyMapper takes as input a set of syntenic regions and a set of one-to-one orthologs between two genomes of interest. Duplications of genes leads to presence of one-to-many and many-to-many co-orthologs, which are converted to one-to-one orthologs using the following pre-processing procedure (see Figure 2):

Genes are grouped into one-to-many and many-to-many ortholog groups
Each one-to-many group is reduced to a single one-to-one ortholog pair with the highest sequence identity
Asymmetric many-to-many groups (those containing *n* genes in the genome *A* and *m* genes in the genome *B*, with 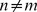) are converted to symmetric many-to-many groups. For example, if 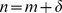, exactly *δ* genes will be removed from the genome *A* based on an ascending ranking according to the average percent sequence identity of each gene to all other genes in the group.
Many-to-many groups, all of which are now symmetric, are split into individual one-to-one orthologous pairs. For any many-to-many group consisting of *n* genes 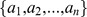 in genome *A* and *m* genes 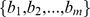 in genome *B* this is achieved by considering only orthology relationships between the genes with the same sequential number (i.e. 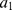 with 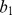, 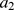 with 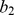, etc.).

This pre-processing procedure aims to identify syntenic orthologs among groups of orthologous genes, i.e. those genes among a set of duplicated genes that are located at equivalent genomic positions with respect to their neighbors. For this reason, gene order is considered as the main factor for resolving many-to-many groups. In one-to-many groups sequence identity is used as a rough measure to identify the most recent ancestor gene.

As an example, Figure 2A shows two syntenic regions, the genes they harbor, and the pairwise orthologous relationships obtained from an external source, such as ENSEMBL [2], [3]. Genes without orthologs (e.g. gene 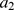) are excluded from consideration. Gene 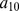 from genome *A* has two orthologs in the genome *B* (one-to-many group). Genes 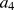, 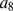 and 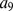 in the genome *A* as well as genes 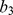 and 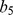 in the genome *B* form asymmetric many-to-many orthologous groups. All remaining genes have only one ortholog. After conducting the pre-processing steps described above, the same syntenic regions look as depicted in Figure 2B. Gene 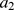 has been removed and gene 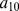 now only has one ortholog with the highest sequence identity (gene 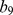). The asymmetric many-to-many group has first been converted to a symmetric one by removing the gene 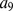, and then split into ortholog pairs according to the gene order, resulting in one-to-one orthologous gene pairs 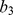-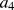
and 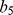-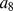.

Given the resulting dataset of one-to-one orthologs, SyntenyMapper identifies two types of evolutionary events - translocation (genes 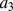 and 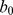 in Figure 2) and inversion of gene order (genes 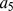, 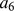, 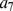, 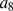 and 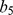, 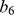, 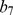, 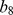). A **translocated** segment is a chromosomal region that breaks off from its original position and reinserts into the genome at another position in one species; during an **inversion** the region inverts its direction before reinsertion. SyntenyMapper finds these events by searching for breakpoints, or locations of unconserved neighborhoods of two genes, in the reference genome *A* by using orthology relationships to genome *B* as a guide.

**Figure 3:**
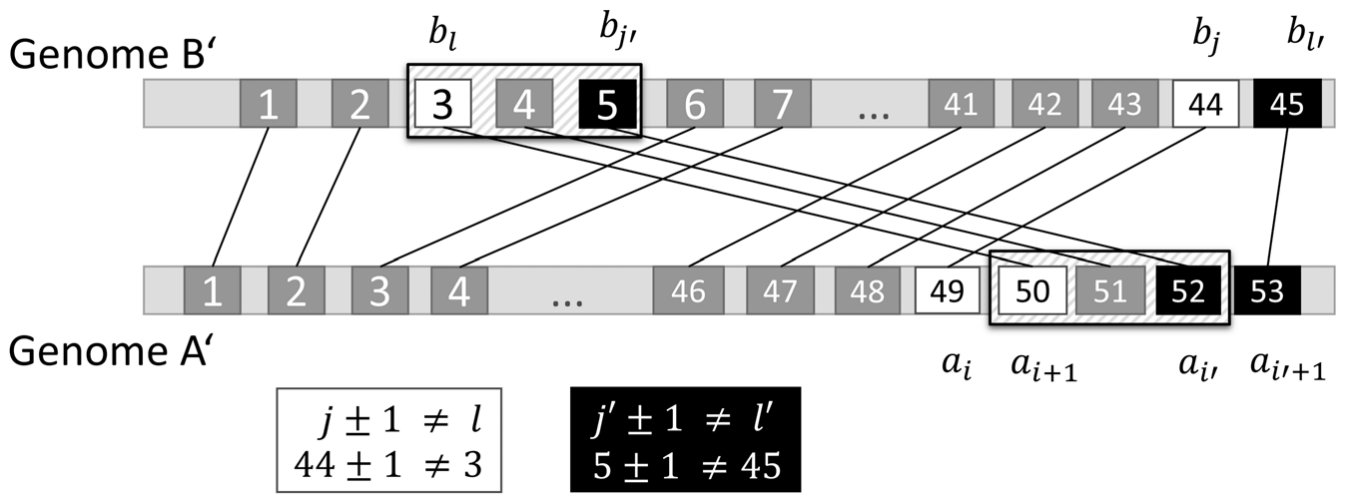
Breakpoint definition in SyntenyMapper. Illustration of two breakpoints emerging at both ends of a translocated segment 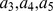 in genome *A* and 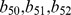 in genome *B* (hatched box). By definition a breakpoint is constituted by two orthologous gene pairs 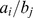 and 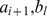 if 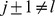, as shown in the boxes underneath the schema. The second breakpoint is described by 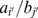 and 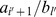. White and black boxes mark the four genes forming the first 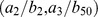 and the second 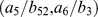
breakpoint, respectively. *A* is used as reference genome to define the block formed by a micro-rearrangement as the genes that lie between the adjacent breakpoints in *A*, in this case 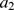, 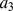 and 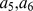. The genes between these two breakpoints and their orthologs in *B* form a block.

A **breakpoint** 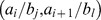 is defined by two orthologous gene pairs 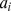, 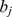 and 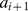, 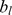 if 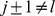, where genes 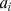, 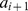 are from the reference genome *A* and genes 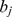, 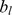 are from the genome *B*. In Figure 2B, breakpoints are marked in both genomes. When a rearranging genomic region reinserts into genome *B* at a new position, **two breakpoints** emerge in *A*, one at each end, as illustrated in Figure 3. Consequently, the genes between two subsequent breakpoints in *A* and their orthologs in *B* either form a so-called **block**, i.e. a set of genes within a translocated or inversed segment, or belong to a non-rearranged part of the original syntenic region that is enclosed between two blocks. To distinguish blocks from syntenic regions with conserved gene order that were not subject to rearrangement, SyntenyMapper looks up the two breakpoints preceding and following 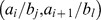 in the genome *B*. If there is no breakpoint preceding and/or following 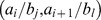, the start and/or end positions of the syntenic region are taken as reference points. The set of these three breakpoints or reference points defines two adjacent segments of genes. The length of both segments is compared and the shorter segment is defined as a new block and a new syntenic region, which lies within the longer original syntenic region.

The choice of a reference genome is arbitrary as each translocated segment in *B* with respect to *A* is also a translocated segment in *A* with respect to *B*. As seen in Figure S1, breakpoint definition with *B* as a reference genome leads to the definition of the same block as was found using *A* as a reference genome (Figure 3).

Within translocated blocks, the direction of genes is identical in both species, while inversed blocks show opposite orientation of genes. The pseudocode of the SyntenyMapper algorithm to find breakpoints and blocks is shown in Figure S2.

In the only step that involves all synteny regions, distant translocations, the so called ‘external orthologs’, are identified by searching for orthologs of those genes without orthologs in their own syntenic blocks. In Figures 2B, C gene 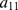 in the syntenic region 1 of the genome *A* and gene 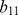 in the syntenic region 2 of the genome *B* do not have orthologs in their own syntenic regions, but are orthologous of each other. These two genes are paired and excluded from their respective syntenic regions to define a new one.

Figure 2C shows syntenic regions refined by SyntenyMapper. Each gene now has a single syntenic orthologous partner while inversed and translocated blocks are redefined as new syntenic regions. Genes or groups of genes that were removed from their syntenic region during one of the prior steps will be termed ‘excluded regions’ in the following sections.

SyntenyMapper is implemented in Java and integrated in the Galaxy platform [18], [19], [20] for easy use and accessibility. Since each syntenic region is treated separately, SyntenyMapper computes micro-rearrangements fast and is able to analyze the human and mouse genomes in under one minute on a standard Linux workstation. Pre-computed results for ENSEMBL Compara syntenic regions data can be downloaded from our website (http://webclu.bio.wzw.tum.de/syntenymapper). The user can also analyze own data using the SyntenyMapper tool within the Galaxy environment if they match the required format described in detail in the help section. Syntenic regions from sources other than ENSEMBL should be defined in a similar manner, i.e. they should represent the longest possible regions evolved from a common sequence in the ancestor. Refined syntenic regions can be visualized using the linear or circular representations implemented in Circos [21] using the tool provided within the SyntenyMapper's Galaxy repository. Additionally, SyntenyMapper generates two output files containing a) coordinates of all syntenic regions, both original and newly defined during the mapping, marked as caused by either an internal or an external micro-rearrangement, and b) syntenic orthologs for each syntenic region.

### TrackMapper

TrackMapper, also implemented in Java, allows users to directly compare so called feature tracks from two species, given a mapping from SyntenyMapper. The tool accepts as input any positional genomic features, such as binding sites or signal intensities, in the generally accepted BED format. In a BED file each line corresponds to one genomic element and contains genomic position (chromosome number, start and end coordinates) as well as an optional signal weight in columns 1–4, respectively. For each gene left after SyntenyMapper preprocessing, TrackMapper calculates the average coverage, *i.e.* the percentage of the gene's base pairs overlapping with the feature (e.g. a LINE repeat) if no signal weight is given, or the average value of the feature if a signal weight is given. These values often represent the strength of a signal, such as the height of a histone modification peak or the expression value. The vector of average coverage values for each gene is then converted into Z-scores by subtracting the mean and dividing by standard deviation. Let 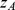 and 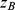 be the vectors of feature Z-scores for syntenic one-to-one orthologs found by SyntenyMapper in genomes *A* and *B*, respectively. The measure of similarity between any two feature tracks, calculated as 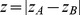, can be downloaded or further analyzed with other Galaxy Tools, which provide a wide range of plotting tools, such as distribution histograms, and statistical analyses, such as correlation tests. Additionally, TrackMapper provides the vector mean as a compact similarity measure between the two tracks over all genes.

Currently, a widely used tool for mapping of tracks between species is LiftOver [22], which was not designed for the purpose of comparing two genomes and is also asymmetrical, in that it converts a feature track from one reference species to the other. By contrast, TrackMapper is able to directly compare feature tracks from two species on the gene level without defining one genome as a source and the other as a target.

### Test data: syntenic regions and orthologs from human and mouse

Data on syntenic regions and orthologs in *Homo sapiens* and *Mus musculus* (assemblies hg19 and mm10, respectively) were obtained from ENSEMBL Compara (version 73) [3]. We downloaded 356 syntenic regions with a mean length of 7.63 mB and 6.89 mB in human and mouse, respectively, and the complete set of ENSEMBL protein-coding genes containing 23,618 and 22,769 unique genes in human and mouse with mean lengths of 59.8 kB and 44.3 kB, respectively. In *Homo sapiens*, an average of 55.10 genes fall into each synteny region, while in *Mus musculus* each syntenic region contains 58.40 genes on average. 27,453 pairwise orthology relationships between protein-coding genes were also downloaded from ENSEMBL.

To test the performance of TrackMapper in analyzing the conservation of genomic feature tracks we obtained ENCODE data for three histone modifications (H3k4me1, H3k4me3, H3k9ac) for human (Broad Institute, embryonic stem cells) and mouse (Ren Lab, ES-E14) [23]. Coordinates were converted to the most recent human (hg19) and mouse (mm10) genome assemblies using LiftOver [22]. For randomization we created *n* elements of the same length as the original peaks at random positions on each chromosome, with *n* being the number of histone modification peaks on this chromosome.

### Calculation of sequence similarity between syntenic regions

We determined sequence similarity between ENSEMBL syntenic regions using our own implementation of the linear runtime trie-based sequence comparison algorithm proposed by Rieck and Laskov [24]. A trie is a tree structure similar to a suffix tree, with the distinction that for a finite set of strings, every string is represented in the path from the root to one of the leaves. Long sequences such as syntenic regions are converted into finite sets of strings by splitting them into overlapping *k*-mers (in our case *k* was set to 6). They are inserted into a trie by iterating over each 6-mers' characters and, starting from the root of the trie, following the edge with the corresponding character to the next node until no such edge exists and one or more new edges need to be inserted.

The algorithm then makes use of the trie's maximum depth of *k* by performing a parallel depth-first search on the tries of the two sequences. A distance score is calculated for each node using the inner function m, which compares the number of occurrences of the string represented by that node in both sequences. The score is accumulated over the whole trie using the outer function ⊕. In our implementation, we used the sum ∑ as outer function ⊕ and the Manhattan distance as inner function *m* (Equation 1).
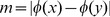
(1)where 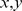 are trie nodes and 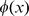 returns the number of occurrences of the string readable from the root of the trie to 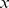 in the sequence.

### Comparison with CYNTENATOR, i-ADHoRe and MCScanX

We compared SyntenyMapper with the latest versions of CYNTENATOR [15] (November 2012), i-ADHoRe 3.0 [17] and MCScanX [16] (May 2012). For each ENSEMBL syntenic region we used as input the actual gene order order and sequence identity values between the corresponding ENSEMBL orthologs. CYNTENATOR was run on each syntenic region with default parameters and homology type BLAST to consider identities, and a simple guide tree containing only human and mouse. We ran i-ADHoRe with the suggested gap size and cluster gap size of 15, probability cutoff 0.001, q-value 0.9, and anchor points 3. For MCScanX, the program MCScanX_h was used with default parameters.

## Results

### SyntenyMapper: a novel tool for refining syntenic orthologs

In this work we propose a new comparative genomics tool for the identification of micro-rearrangements within syntenic regions that were shaped by macro-rearrangements between species. SyntenyMapper complements the set of one-to-one orthologs from ENSEMBL by finding syntenic one-to-one orthologs among one-to-many/many-to-many orthologous groups and uses them to identify deletions, inversions, local and distant translocations within syntenic regions, further refining the definition of these regions. As a result, SyntenyMapper provides the user with a set of evolutionary building blocks with completely conserved gene order between two species.

The tool is easily accessible through the Galaxy Platform and can be used to analyze both user-supplied syntenic regions as well as those obtained from ENSEMBL Compara. The resulting refined syntenic regions and their associated annotation tracks can be interactively visualized using the circular Circos representation [21] or a linear plot created with an R script (for an example see Figure S3), and analyzed with our own track-comparison tool called TrackMapper.

### Detection of micro-rearrangements between human and mouse genomes

To illustrate the performance of the SyntenyMapper approach, we applied it to syntenic regions and orthologs between the human and mouse genome obtained from ENSEMBL Compara. Syntenic region length is highly correlated between these species (Figure S4).

As seen in Table 1, the majority of protein-coding ENSEMBL genes (70.07%) have exactly one ortholog within their syntenic region. The second largest subset (12.30%) is constituted by those genes that do not have any orthologs and are hence not treated by SyntenyMapper. Genes in many-to-many groups within one syntenic region comprise only 2.30% of the total gene number; most of these groups are asymmetric. Finally, genes only having orthologs in another syntenic region (external orthologs) make up a mere 1.27% of the data. All other types of orthologous relationships, such as genes with orthologs in regions not covered by syntenic regions, represent less than 2% of all cases and are ignored by the current version of SyntenyMapper.

**Table 1:** Frequency of different cases of orthologous relationships for a given gene in a syntenic region between human and mouse.

Type of orthologous relationship	#Genes
*No ortholog*	5,705 (12.30%)
*One internal ortholog*	32,505 (70.07%)
*Many internal orthologs*	1,062 (2.29%)
*One external ortholog*	487 (1.05%)
*Many external orthologs*	105 (0.23%)
Many in- and external orthologs	204 (0.44%)
One ortholog in a syntenic-block-free region	99 (0.21%)
Many orthologs in a syntenic-block-free region	15 (0.03%)
Many orthologs: internal, external, and in syntenic-block-free regions	166 (0.36%)

Italic font indicates classes of genes that are covered by SyntenyMapper. Internal orthologs: Orthologous genes that lie in the same syntenic region. External orthologs: Orthologous genes that lie in different syntenic regions. Syntenic-block-free region: Genomic region that is not covered by ENSEMBL syntenic regions.

During the rearrangement detection process, SyntenyMapper implements a pre-processing step to find syntenic ortholog pairs among the set of orthologous genes. While converting one-to-many and many-to-many ortholog groups into one-to-one orthologs, a total of 941 genes (2.1% of all human and mouse genes) are removed as non-syntenic. As all relationships of these genes are non-syntenic as well, a total of 10,840 relationships from the original set of 27,453 ENSEMBL Compara orthologs are eliminated with the non-syntenic genes that formed them.

SyntenyMapper finds 16,613 syntenic ortholog pairs between human and mouse and detects 2,898 blocks that were subject to micro-rearrangements within or between the original 356 syntenic regions. There are three different types of syntenic regions obtained after this analysis: i) original regions, which are identical to the input regions except for excluded non-syntenic genes (356, 10.94%), ii) internally translocated regions that represent blocks created by micro-rearrangements within the syntenic region (2,817, 86.57%), and iii) externally translocated regions that represent one or more neighboring genes that were translocated between syntenic regions (81, 2.49%).

Most frequent are internal micro-rearrangements, but blocks caused by these contain only 1.52 genes on average, while the majority of genes (73.68%) still lie within original syntenic regions. We can thus conclude that often blocks consisting of just one or two genes disturb the conservation of gene order within a syntenic region. Regions translocated over large distances are rare and, in the specific case of human/mouse comparison presented here, almost always contain only a single gene. Genes within one syntenic region lie within a linear distance of around 7 to 8mB from each other. Hence, the proximity of genes within a syntenic region leads to more frequent rearrangements compared to large linear distances. Linear proximity correlates with spatial proximity, which has been shown to be one of the triggers for rearrangements [25].

One major advantage of our approach is its ability to refine regions formed by macro-rearrangements, which are large-scale intra- or inter-chromosomal translocations, through the consideration of all micro-rearrangements, whereby short segments change their positions within syntenic regions [26]. SyntenyMapper uses pre-calculated syntenic regions, for example from ENSEMBL, which are created in such a way that their length is maximal, disregarding small rearrangements within them (see introduction). Identification of micro-rearrangements can greatly improve our understanding of evolutionary events shaping extant genomes and is instrumental for comparing the properties of regions of conserved gene order between two species.

**Figure 4:**
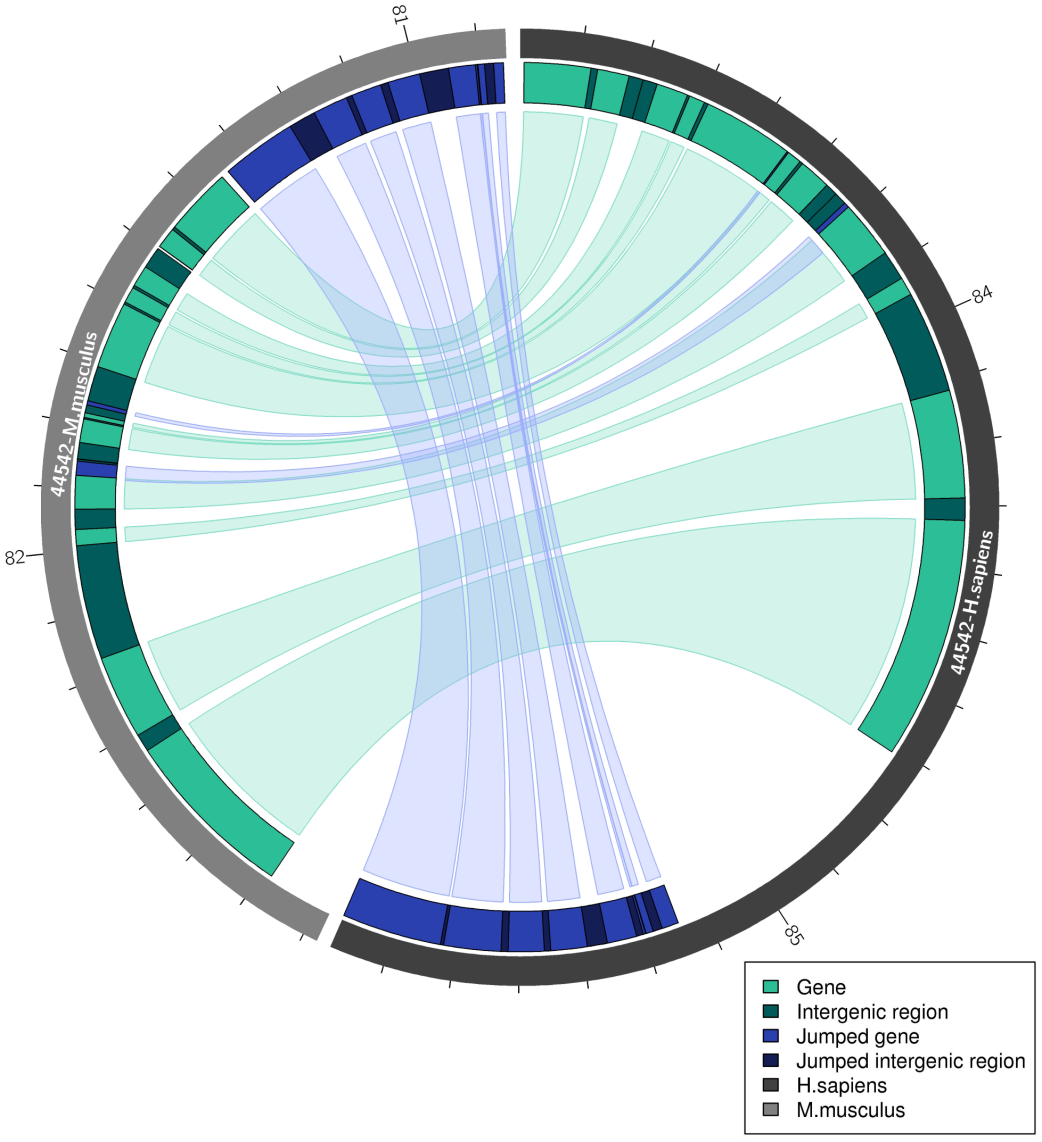
Visualization of SyntenyMapper results for a syntenic region in human and mouse. The region (ENSEMBL identifier 44542) is illustrated in human as a dark grey ideogram (right) and in mouse as a light grey ideogram (left). Ticks are placed at 100 kB distance and the numbers represent positions in mB on the human and mouse chromosomes 15 and 7, respectively. The Circos circular plot illustrates the positions of genes/intergenic regions for one syntenic region in both species and the correspondence between them. Micro-rearrangements are illustrated by color-coding, with syntenic orthologs and out-of-order genes shown in grey and black, respectively, while the intergenic regions between syntenic orthologs and between out-of-order genes are shown in white. A large block of seven genes (black) was translocated in either human or mouse. In the Galaxy version of the plots, gene annotations are given as labels and as direct links to ENSEMBL through clicks onto the gene track.

Figure 4 shows a syntenic region with the same orientation in human and mouse, with most of the genes having syntenic one-to-one orthology relationships. However, the order of genes is disrupted by a translocation of a large block containing seven genes located in the beginning of the human region and in the end of the mouse region. The order and direction of genes within this block is preserved. Interestingly, there is a sizeable gap between syntenic orthologs and translocated genes on the human chromosome, which may imply that the translocation happened in *Homo sapiens* after human-mouse divergence.

SyntenyMapper is also able to identify a small-scale rearrangement with the majority of genes within the syntenic region in mouse lying on the reverse strand, even though the region is annotated as situated on the forward strand (Figure S5). In human, all genes lie on the forward strand, implying that an inversion occurred in mouse. Our approach recognizes this inversion of multiple genes and redefines it as a new syntenic region.

These examples illustrate the difficulties in comparing genome regions that evolved from the same sequence due to recent genome rearrangements that make any linear comparison impossible. Our method enables the user to refine syntenic regions and to discover micro-rearrangements at the level of individual genes. The analysis is not restricted to translocations within one region and also considers those genes that lie within other syntenic regions.

As an example, Figure S6 shows the relationship between two pairs of syntenic regions that share one orthologous gene pair. These rare external translocations (592, 1.27% of all genes) of genes out of their syntenic region almost always involve single genes in the human/mouse comparison. We hypothesize that translocations of single genes over large linear distances are favored by short spatial distances between the chromosomal regions involved, as has been shown for cancer cells [25]. All rearrangements illustrated in this section were not found by ENSEMBL Compara and cannot be directly inferred from orthology relationships, stressing the value of SyntenyMapper for comparative genomics.

To demonstrate the usefulness of TrackMapper for comparing feature tracks between genomes analyzed histone modifications, which are known to be largely conserved between mouse and human [27]. Using histone modification data from ENCODE we applied TrackMapper to calculate the measure that quantifies the difference in the extent to which genes overlap with histone modification peaks in human and mouse. For all three histone modifications considered here (see *Methods*) the difference measures are low, with a respective mean of 0.77, 0.63 and 0.70 for H3k4me1, H3k4me3 and H3k9ac. We ran TrackMapper on mouse data and randomized human histone modification peaks, to receive significantly higher difference value distributions (Wilcoxon rank sum test p-value <2.2e-16 in all three cases) with means of 0.98, 1.00 and 1.02, respectively. Of special interest are genes with very low difference scores, which can be easily identified using TrackMapper's tabular output (File S1). There are 171 ortholog pairs with a very low H3k4me3 difference score (below 0.01). TrackMapper is thus a convenient tool for in-depth analysis of feature conservation at the gene-level.

### Pre-computed ENSEMBL Compara species comparison

ENSEMBL Compara provides syntenic regions and orthologs for 25 pairs of eukaryotic species, with 16 of them involving human. We conducted a large-scale analysis of micro-rearrangements within these syntenic regions using SyntenyMapper and calculated the number of micro-rearrangements within and between syntenic regions as well as the average number of genes involved in these events (Table S1).

Among the most closely related species pairs are human *vs* chimp (*P. troglodytes*) and mouse *vs* rat (*R. norvegicus*). Both of them share a high number of orthologs, however, the number of ENSEMBL syntenic regions differs vastly: while human and chimp share only 139 syntenic regions, there are 554 ones between mouse and rat. Because the average syntenic region length between human and chimp is about four times the size of syntenic regions in mouse and rat (22mB and 4.8mB, respectively), the percentage of the genomes covered is similar (human *vs* chimp: 87.76%, mouse *vs* rat: 94.64%).

**Figure 5:**
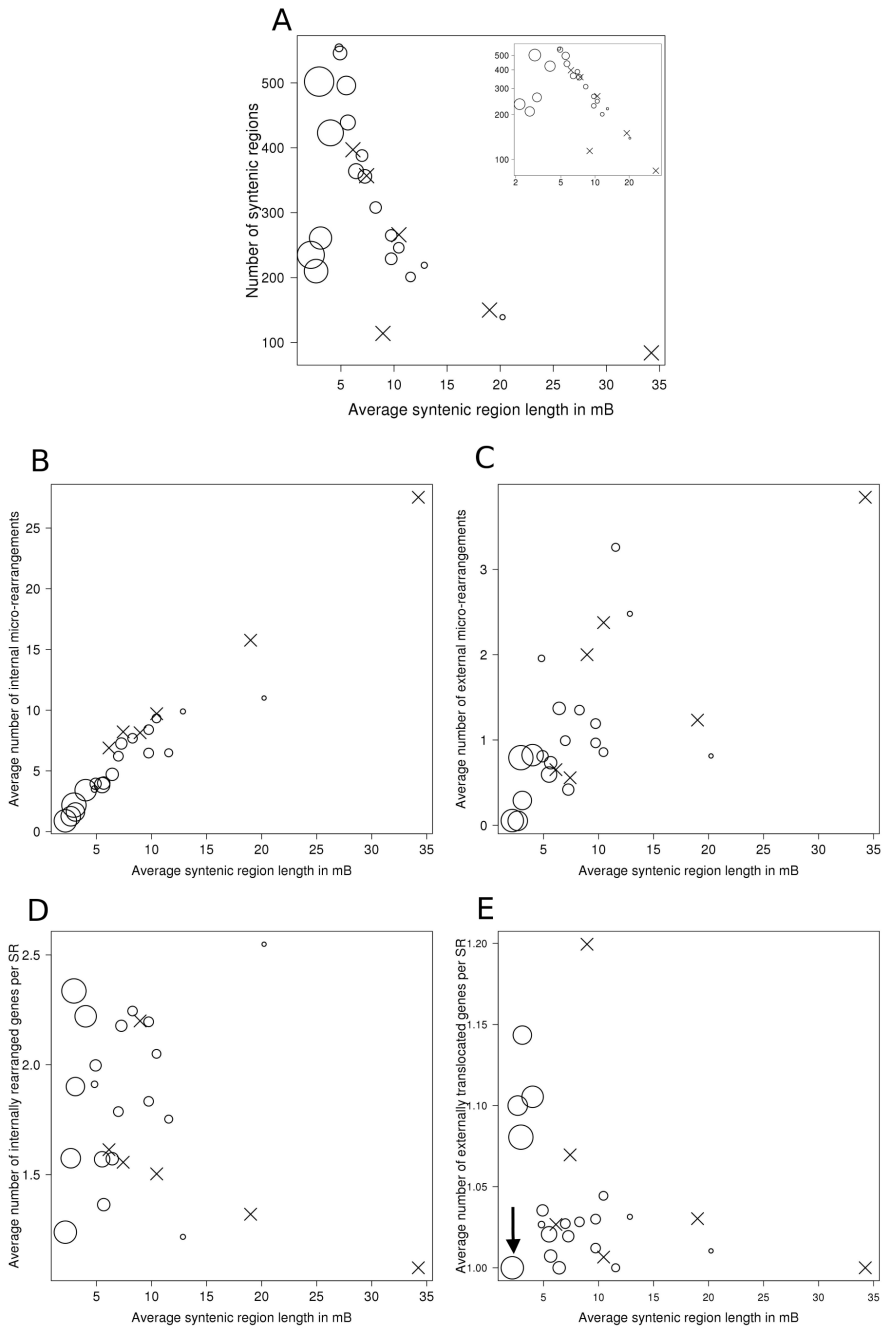
Relationship of syntenic region length, evolutionary distance and other features. Dependence of synteny features on the average syntenic region length (x axis) and evolutionary distance (circle size, inferred from branch lengths in Miller et al (2007), calculated as the average number of substitutions per site). Crosses correspond to the species pairs with no distance information available. A) Negative exponential correlation between the number of syntenic regions and their average length (inset: logarithmic axis scales). Closely related species (small circles) tend to have fewer, longer syntenic regions. Distant species (large circles) tend to have high numbers of very short syntenic regions. B) The average number of internal micro-rearrangements per syntenic region strongly correlates with syntenic region length and evolutionary distance. Evolutionary distant pairs of species share short syntenic regions with few internal micro-rearrangements. C) No clear correlation between syntenic region length and rearrangement number can be observed for external micro-rearrangements. D) The size of internal micro-rearrangements (average number of genes involved) does not correlate with the syntenic region length and evolutionary distance. E) Similarly, the number of genes involved in external translocations is generally independent of the syntenic region length. However, with the exception of mouse and platypus (average number of genes in externally translocated regions: 1.0, marked by arrow), distant species pairs tend to have somewhat longer externally translocated regions (average>1.05).

Using branch lengths of the UCSC species tree [28] (available for all species pairs considered in this study except for those involving gorilla, pig, orangutan, common marmoset and turkey) we analyzed the correlation between the evolutionary distance and micro- and macro-rearrangement related genome features. In general, one would expect more closely related species to share a low number of very long syntenic regions, and an increase in the number and a decrease in the average length of syntenic regions with the growing evolutionary distance. Indeed, for species pairs separated by short or medium evolutionary distance, this expectation is true and the average syntenic region length and their number exhibit a negative exponential correlation (Figure 5A). However, more distantly related species, such as human and platypus, contain fewer ENSEMBL syntenic regions than would be expected based on this exponential correlation pattern. Strong deviation from this correlation pattern observed for the species pairs involving platypus as well as in the lizard vs chicken comparison is caused by the very low sequence similarity between these species, which leads to a low coverage of the genomes by syntenic regions (e.g. human *vs* platypus: only 21% of the genome is covered, see Table S2). Two other distant species pairs - human *vs* chicken and mouse *vs* chicken – are more in line with the general trend, but due to significant differences in genome size (human genome 3.1 gB, mouse genome 2.7 gB, chicken genome 1.1 gB), they also show a low genome synteny coverage of the respective longer genomes.

Because of SyntenyMappers's gene-based approach, the number of detected internal and external micro-rearrangements is dependent of the number of orthologs, which is higher for closely related species. As seen in Figure 5B, the average number of internal micro-rearrangements (per syntenic region), ranging between 0 and 30, increases with the increasing average syntenic region length and decreasing evolutionary distance. This implies that the number of micro-rearrangements depends on the size of the syntenic regions that harbor them, which is in turn correlated with evolutionary distance, as illustrated in Figure 5A.

While the total number of micro-rearrangements indirectly depends on the degree of relationship between the genomes, there is no correlation between the number of micro-rearrangements (internal or external) per mB and evolutionary distance (Figure S7). We also found only a slight positive correlation between sequence distance and the number of internal micro-rearrangements (Pearson correlation coefficient 0.22). In particular, low sequence similarity does not necessarily lead to a high number of micro-rearrangements (Figure S8).

The higher numbers of micro-rearranged regions per syntenic region in more closely related species are thus mainly due to the greater length of syntenic regions. By contrast, the size of micro-rearranged regions (i.e. the number of genes they contain) does not show any dependence on the syntenic region length and the evolutionary distance (Figure 5C), because the mechanisms of transposition, which is the main cause of small-scale translocations, are the same in all species.

External translocations between syntenic regions are less common, with the average number per syntenic region ranging between 0 and 4 for all species pairs. Overall there is a slight tendency for longer syntenic regions in more related species to contain a higher number of such external micro-rearrangements (Figure 5D). In almost all cases, only a single gene is subject to an external micro-rearrangement, regardless of the size of the synteny regions or the evolutionary distance (Figure 5E). However, with the exception of mouse and platypus, which harbor on average only 1.0 external micro-rearranged genes per syntenic region, all distantly related species pairs (mouse vs chicken, human vs platypus, human vs chicken, chicken vs lizard) have somewhat longer external translocations (average>1.05). Larger external micro-rearrangements (1.3 genes in external micro-rearrangements per syntenic region) are especially common between chicken (*G. gallus*) and wild turkey (*M. gallopavo*). In particular, SyntenyMapper was able to detect a group of six consecutive genes that was translocated between two syntenic regions in these organisms (see Figure S9). As the only closely related species pair with sharing relatively large external translocations, chicken and turkey genomes behave very differently in this aspect. Cases like this can be easily identified with SyntenyMapper, allowing for detailed genome comparison and further analysis based on the produced mapping.

### Comparison with existing methods

SyntenyMapper is the first tool that was developed to detect micro-rearrangements in the form of blocks of conserved gene order within macro-rearranged regions. However, available software for detection of collinear blocks (i.e. sets of genes with conserved order in two or more species) can be applied to ENSEMBL syntenic regions as well, although no rearrangements between syntenic regions can be detected using this approach.

We detected collinear blocks between human and mouse with SyntenyMapper (discarding single-gene regions), CYNTENATOR, i-ADHoRe and MCScanX.

CYNTENATOR applies the Smith-Waterman algorithm to genomes represented by strings of genes, with homologies provided in the form of BLASTP output, and identifies local alignments, which represent blocks of conserved gene order in both species. The main advantage of this method is its handling of multiple genomes, when provided with a guide phylogenetic tree that can be obtained from other sources.

Since there is no gold standard annotation of syntenic regions, we have to rely on a qualitative comparison of the results. We have identified several reasons for discrepancies between CYNTENATOR and SyntenyMapper:

CYNTENATOR is not designed to detect external micro-rearrangements when applied to syntenic regions. However, this makes up for only a small percentage of the discrepancies: in only 8 of 356 syntenic regions (2.25%) CYNTENATOR misses just the external rearrangements. A larger problem is that CYNTENATOR, in contrast to our method, is unable to detect inversed collinear blocks of any size. Such inversions are common and occur in 170 out of 356 (47.75%) of all syntenic regions, including cases where the complete region is inversed in the second species (see Figure S10 for an example). In contrast, we have identified only one syntenic region where SyntenyMapper misses a collinear block detected by CYNTENATOR because two genes in this block overlap.

Other minor differences affect only the length of collinear blocks and are mainly caused by different definitions. Due to the usage of the Smith-Waterman alignment algorithm for aligning gene strings, CYNTENATOR sometimes mismatches non-orthologous genes, and includes gaps when genes without orthologs occur. SyntenyMapper, while also allowing for the presence of genes without orthologs, will fragment a collinear block that is disrupted by a pair of non-orthologs into two blocks. Additionally, overlapping genes are excluded by our method. When we compare results from CYNTENATOR and SyntenyMapper, we thus often find apparently shorter but equivalent collinear blocks in the results from our method.

Another minor difference is caused by different definitions of collinear blocks. Our definition of a refined syntenic region provides for rearrangements that are embedded within a backbone of conserved gene order that ideally spans the whole syntenic region. In such a case, SyntenyMapper defines two collinear blocks, a small one embedded within a larger one, while CYNTENATOR will define three consecutive blocks.

Furthermore, CYNTENATOR fragments a large collinear block into two blocks if it is disturbed by external single-gene rearrangements, sometimes for no apparent reason (see Figure S11 for an example). SyntenyMapper is thus better suited for identifying all collinear blocks in a syntenic region, including inversions.

Another widely used tool for genome comparison is i-ADHoRe, which detects collinearity on the basis of a gene homology matrix. Blocks of conserved gene order are identified as dense diagonals and the corresponding genes are then aligned using the Needleman-Wunsch algorithm, a greedy graph strategy or a new algorithm named GG2. In this process, gaps are introduced if necessary to produce blocks with as many homologous pairs as possible. The software is well suitable for multi-genome comparisons.

Like CYNTENATOR, i-ADHoRe can be applied to syntenic regions and produces results that are comparable with SyntenyMapper. The main difference between the methods is the insertion of gaps and mismatches in i-ADHoRe alignments to create longer blocks, while our method creates a separate block if it is disrupted by a non-orthologous gene pair. The design goal of i-ADHoRe to detect collinear blocks that contain as many orthologs as possible comes at the expense of frequently missing small rearrangements that are partially embedded in larger ones.

For example, in a syntenic region comprising 11 genes, three of which are translocated within the region, SyntenyMapper correctly identifies the translocation (see Figure S12) while i-ADHoRe ignores it and defines it as a gap. Overall, both methods detect the same amount of collinear blocks in most syntenic regions (222, 62.36%), although mismatches in i-ADHoRe can result in longer detected regions. In the majority of the remaining cases (68, 19.10%) i-ADHoRe misses one or more regions due to mismatches (as described above) or inability to detect external micro-rearrangements.

We have investigated cases where SyntenyMapper detects fewer collinear blocks than i-ADHoRe (43, 12.08%). Besides the implementation differences, where our tool defines a backbone of genes with embedded micro-rearrangements while other tools define multiple adjacent blocks, we have also found that i-ADHoRe tends to disrupt collinear blocks if they are interrupted by a longer stretch of genes without orthologs. By contrast, SyntenyMapper ignores genes without orthologs when defining blocks of conserved gene order. From these results we conclude that, while both methods provide a similar functionality and largely consistent results, SyntenyMapper is better suited for a very detailed analysis of genome rearrangements that requires exact definitions of conserved gene order blocks, without mismatches and gaps. i-ADHoRe and CYNTENATOR, on the other hand, are applicable to more than two genomes and can provide a good overview of less stringent collinear blocks.

MCScanX is another software package that includes not only methods for the identification of collinear blocks. Like CYNTENATOR it requires BLASTP output or a list of orthologous genes as input, and uses a dynamic programming approach to find chains of collinear gene pairs in the two genomes. Its main advantage is the large set of downstream analysis tools, which includes four different visualization modes for the results, classification of duplicated genes into specific classes, and detection of whole genome duplication events.

When investigating reasons for discrepancies between SyntenyMapper and MCScanX, it became apparent that the latter method fails to handle duplicates correctly. For a one-to-many ortholog group it appears as if MCScanX detects multiple overlaying collinear blocks, where a single gene is contained in more than one such block and paired with different orthologs. SyntenyMapper uses a pre-processing procedure based on gene order and sequence similarity to identify one-to-one orthologs among sets of duplicated genes. As a consequence, each gene is associated with a single gene in the other genome, allowing for overlap-free definition of collinear blocks. MCScanX appears to keep one-to-many or many-to-many groups and defines blocks with genes in conserved order regardless of whether or not these genes are already present in other blocks. This behavior leads to significant overlapping between collinear blocks.

Per definition, a single gene cannot be part of two or even more collinear blocks. From these results we conclude that MCSCanX cannot provide the same functionality as SyntenyMapper, CYNTENATOR or i-ADHoRe.

## Discussion

SyntenyMapper is a new rapid tool for comparative genomics that enables analysis of micro-rearrangements, detection of positional orthology, and direct comparison of genomic features between two species. Previously developed methods focus on finding regions caused by either macro- or micro-rearrangements on a global scale and use either whole-genome alignments or homologous elements alone to define these. By contrast, we identify micro-rearrangements within large synteny regions by focusing on the conservation of gene order. This combination of alignments and homologous elements allows us to find large blocks with conserved gene order that can be seen as the smallest evolutionary building blocks within largely conserved regions. The analysis of these numerous internal rearrangements can provide additional insights into evolutionary history and serves as a better basis for comparative genomics analyses than the original syntenic regions alone.

We have applied SyntenyMapper to 25 species pairs using syntenic regions and ortholog sets from ENSEMBL Compara. As expected, closely related species contain few but long syntenic regions, compared to a high number of short regions in distant pairs. The number of internal micro-rearrangements is proportional to the size of the synteny region they reside in, which in turn depends on the degree of relationship between the genomes. However, the density of micro-rearrangements, i.e. the number of micro-rearrangements per mB, does not correlate with the evolutionary distance. We found that similar sequences tend to have fewer micro-rearrangements, but the correlation is weak.

More distant species pairs tend to have short to medium syntenic regions harboring between 0 and 10 internal micro-rearrangements while more closely related organisms with long syntenic regions (over 10 mB length) contain between 10 and 25 micro-rearrangements. External syntenic regions are rare, with only 0 to 4 such translocations per syntenic region in all genome pairs, and show only a slight trend for a higher number in longer regions. We speculate that external translocations occur only under very specific circumstances, when distant genome regions are spatially close.

There is no correlation between the number of internally translocated genes in a micro-rearranged block and syntenic region length or evolutionary distance, implying that the transposition mechanism acts regardless of evolutionary history. While macro-rearrangements arise by complex processes involving chromosome double strand breaks [25], small-scale rearrangements are most likely caused by cut-and-paste DNA transposition [29]. Our results show that there are constraints that limit the total length of the translocated genome region. DNA transposons are often used as vector elements in biotechnology, and it has been reported that the efficiency of transposition decreases with increasing cargo size [30], [31], [32]. We show that the average cargo of transposons in higher eukaryotic genomes comprises between 1 and 2.5 genes for translocations over short linear distances, and between 1 and 1.2 genes for more distant translocations. This implies that the length constraints of transposable elements are also dependent on the linear distance between the source and the target position in the genome. This insight could be valuable for biotechnology, where ways to overcome the length limitations in transposons are searched for [32], [33]. External translocations are also not correlated with the evolutionary distance or syntenic region length, but longer externally translocated regions appear to occur only in distant species pairs.

In general, we conclude that syntenic regions between the genomes of closely related species are longer and contain more micro-rearrangements of genes due to their length. More distant species pairs contain a higher number of short and less similar syntenic regions with fewer micro-rearrangements. The density and size of these is largely independent of the evolutionary distance and only slightly influenced by sequence similarity.

SyntenyMapper creates a one-to-one mapping of genes between species similar to positional orthology methods. Based on this mapping, TrackMapper is able to directly compare quantitative and qualitative features of genes, such as values representing the relative time point of replication or the overlap with long terminal repeats, and calculate a similarity measure for each pair of orthologs and the complete set of genes.

Beyond comparative genomics, identification of micro-rearrangements may be instrumental in understanding disease mechanisms. A number of diseases caused by micro-rearrangements are known, including the DiGeorge syndrome or Miller-Dieker lissencephaly [34].

## Supporting Information

Figure S1
**Effect of reversing the reference genome used in the example shown in** **Figure 5** **.** The reference genome used here for the definition of breakpoints is A′ ( = B in Figure 5). The detected breakpoints are 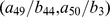 (white boxes) and 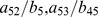 (black boxes). Based on the adjacent breakpoints in the new reference genome A′, the same translocated segment (hatched box) is detected as in Figure 2.(PNG)

Figure S2
**Pseudocode of the SyntenyMapper algorithm to find rearranged segments.** SyntenyMapper uses externally defined syntenic regions and one-to-one orthologs as input to find maximum length blocks of conserved gene order (termed blocks). It iterates over all genes in genome A and identifies breakpoints according to the definition given in the *Methods* section, using the helper function 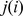, which returns the index of gene 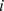
's ortholog in genome B. SyntenyMapper identifies the two breakpoints preceding and following the detected breakpoint 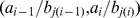, because each pair of adjacent breakpoints encloses either a rearranged genomic segment or the region between two rearranged segments, as described in *Methods*. To distinguish these two cases, lengths of both segments defined by the three adjacent breakpoints are compared and the shorter of the two is defined as a block resulting from a micro-rearrangement with respect to the longer original syntenic region. The type of the rearrangement (translocation or inversion) is detected based on gene order within this block.(PNG)

Figure S3 **Linear representation of a syntenic region (ENSEMBL identifier 44542) produced by SyntenyMapper.** Upper half shows genes in mouse, lower half genes in human. Green genes are those with conserved order, translocated or overlapping genes are colored blue. To better see these overlapping genes, conserved order and translocated genes are depicted in independent lines and a combined line, marked in the legend on the right and left edge of the plot.
(PNG)

Figure S4 **Comparison of syntenic region length in human and mouse.** The regression line is shown as dotted line (Pearson correlation coefficient 0.9932).
(JPG)

Figure S5 **A syntenic region (ENSEMBL identifier 44514) in human (dark grey) and mouse (light grey) containing a large inversed segment, illustrated in blue and only one ortholog with the same orientation as the syntenic region (green).** Ticks are placed at 100 kB distance and the numbers show the position on chromosomes 13 (human) and 14 (mouse) in mB.
(PNG)

Figure S6 **Translocation of a single gene from the human region 44801 to the mouse region 44598, shown with red line.** Ticks are placed at 100 kB distance and the numbers show the positions in mB on chromosomes X in human and mouse (region 44801) as well as on chromosomes 19 in human and 7 in mouse (region 44598).
(PNG)

Figure S7 **Average number of micro-rearrangements (internal and external) per megabase covered by syntenic regions versus evolutionary distance.** No correlation can be observed.
(PNG)

Figure S8 **Syntenic regions (SR) with low sequence similarity (high distance score) show a trend to contain more internal micro-rearrangements per megabase (Pearson correlation coefficient 0.22).** Outliers with extremely high sequence distance are not shown.
(JPG)

Figure S9 **A translocation involving six consecutive genes between two syntenic regions in chicken (** ***G. gallus*** **, dark grey) and wild turkey (** ***M. gallopavo*** **, light grey), marked by red lines.** Ticks are placed at 100 kB distance and the numbers show the positions in mB on chromosomes 6 in chicken and 8 in turkey (region 47198) as well as on chromosomes 17 in chicken and 19 in turkey (region 47135).
(PNG)

Figure S10 **An example of an inversed syntenic region between human and mouse where CYNTENATOR detects no collinear block despite eleven genes lying in the same order in both genomes.**
(PNG)

Figure S11 **An example of a syntenic region between human and mouse where CYNTENATOR fragments the existing collinear block into two blocks (location of split marked red in both species) despite lack of genes disrupting the gene order.**
(PNG)

Figure S12 **Example of a syntenic region (ENSEMBL identifier 44459) including a micro-rearrangement of three genes that is correctly identified by SyntenyMapper but ignored as a gap by i-ADHoRe.**
(PNG)

Table S1 **Statistics of pre-computed synteny mapping for ENSEMBL Compara (version 73).**
(PDF)

Table S2 **Evolutionary distance and genome coverage by syntenic regions for all species pairs.** SR: Syntenic region.
(PDF)

File S1 **List of human and mouse orthologs with significantly high similarity in H3k4me3 peak density (TrackMapper difference score below 0.01).**
(TXT)
