# Supplementary figures and images for: Analysis of Micro-Rearrangements in 25 Eukaryotic Species Pairs by SyntenyMapper
Source: PLoS One. 2014 Nov 6;9(11):e112341. doi: 10.1371/journal.pone.0112341 (PMC4223023; doi:10.1371/journal.pone.0112341)

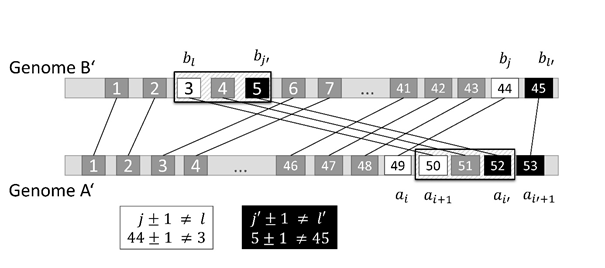

Supplement: Figure S1 — Effect of reversing the reference genome used in the example shown in Figure 5 . The reference genome used here for the definition of breakpoints is A′ ( = B in Figure 5). The detected breakpoints are (white boxes) and (black boxes). Based on the adjacent breakpoints in the new reference genome A′, the same translocated segment (hatched box) is detected as in Figure 2. (PNG) [file pone.0112341.s001.png]

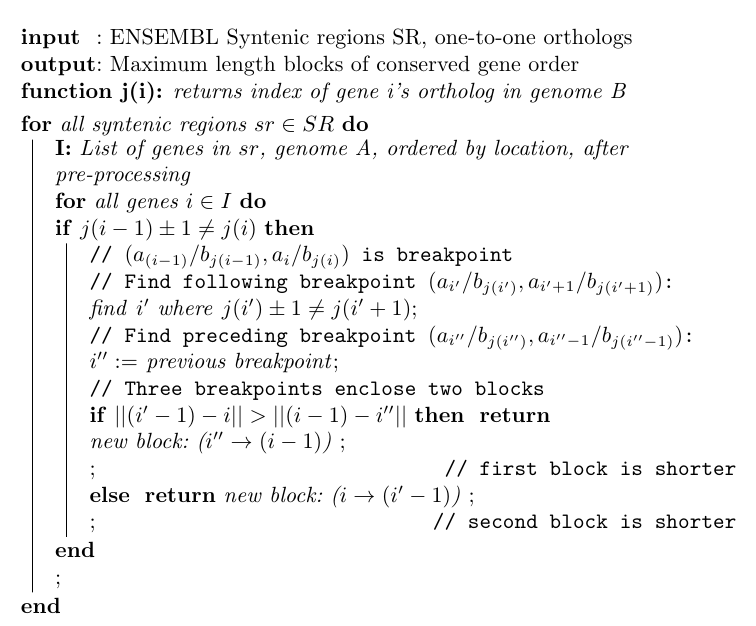

Supplement: Figure S2 — Pseudocode of the SyntenyMapper algorithm to find rearranged segments. SyntenyMapper uses externally defined syntenic regions and one-to-one orthologs as input to find maximum length blocks of conserved gene order (termed blocks). It iterates over all genes in genome A and identifies breakpoints according to the definition given in the Methods section, using the helper function , which returns the index of gene 's ortholog in genome B. SyntenyMapper identifies the two breakpoints preceding and following the detected breakpoint , because each pair of adjacent breakpoints encloses either a rearranged genomic segment or the region between two rearranged segments, as described in Methods . To distinguish these two cases, lengths of both segments defined by the three adjacent breakpoints are compared and the shorter of the two is defined as a block resulting from a micro-rearrangement with respect to the longer original syntenic region. The type of the rearrangement (translocation or inversion) is detected based on gene order within this block. (PNG) [file pone.0112341.s002.png]

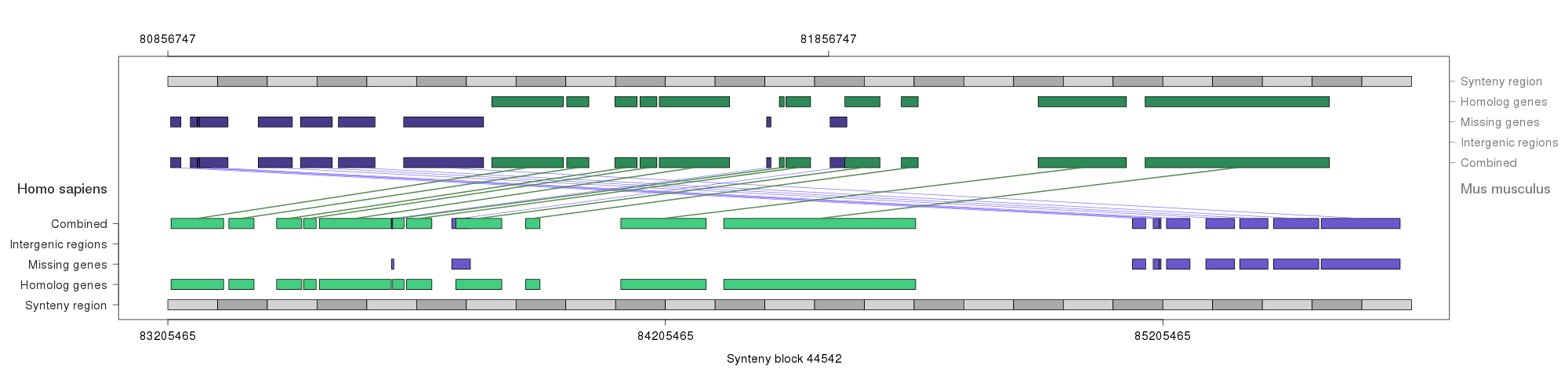

Supplement: Figure S3 — Linear representation of a syntenic region (ENSEMBL identifier 44542) produced by SyntenyMapper. Upper half shows genes in mouse, lower half genes in human. Green genes are those with conserved order, translocated or overlapping genes are colored blue. To better see these overlapping genes, conserved order and translocated genes are depicted in independent lines and a combined line, marked in the legend on the right and left edge of the plot. (PNG) [file pone.0112341.s003.png]

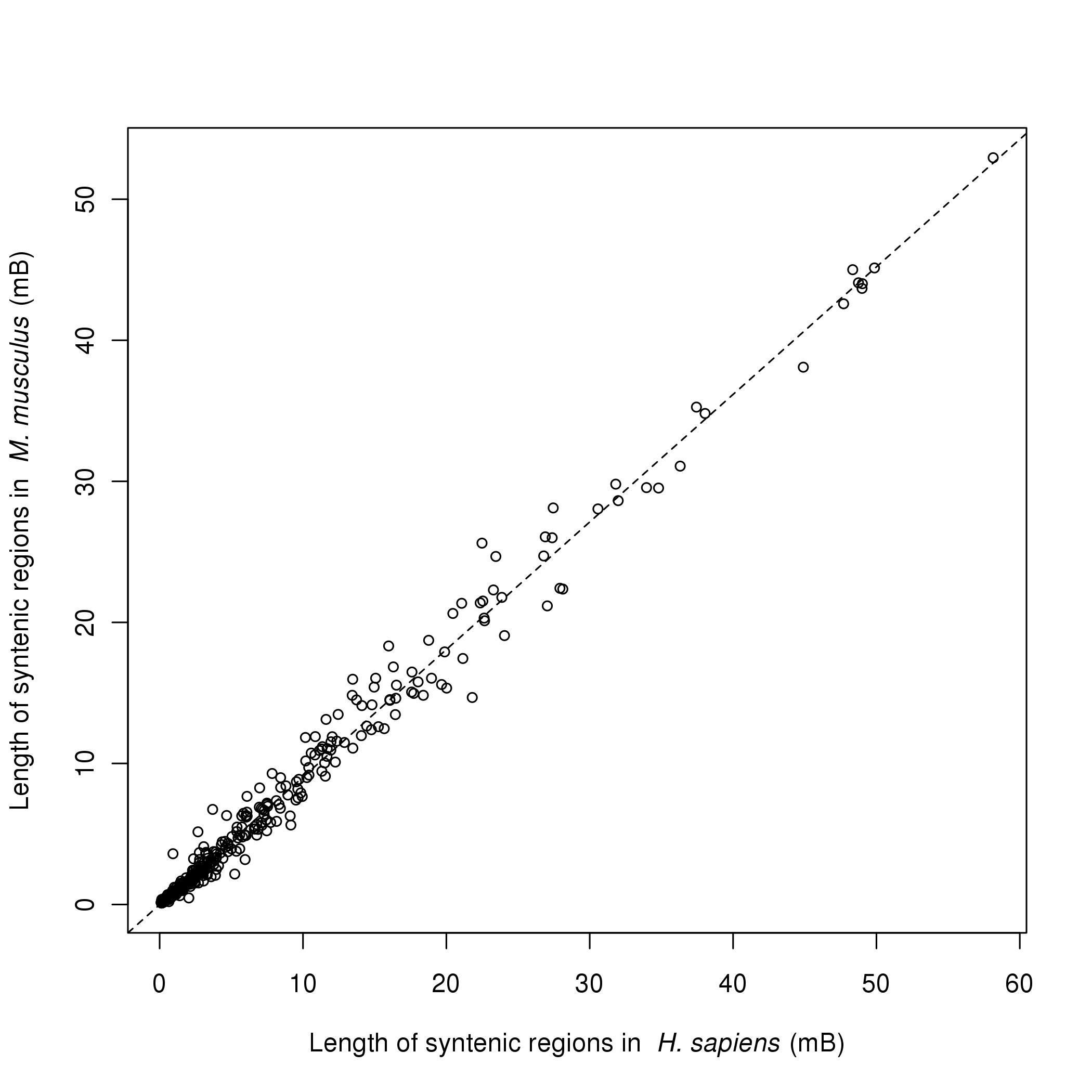

Supplement: Figure S4 — Comparison of syntenic region length in human and mouse. The regression line is shown as dotted line (Pearson correlation coefficient 0.9932). (JPG) [file pone.0112341.s004.jpg]

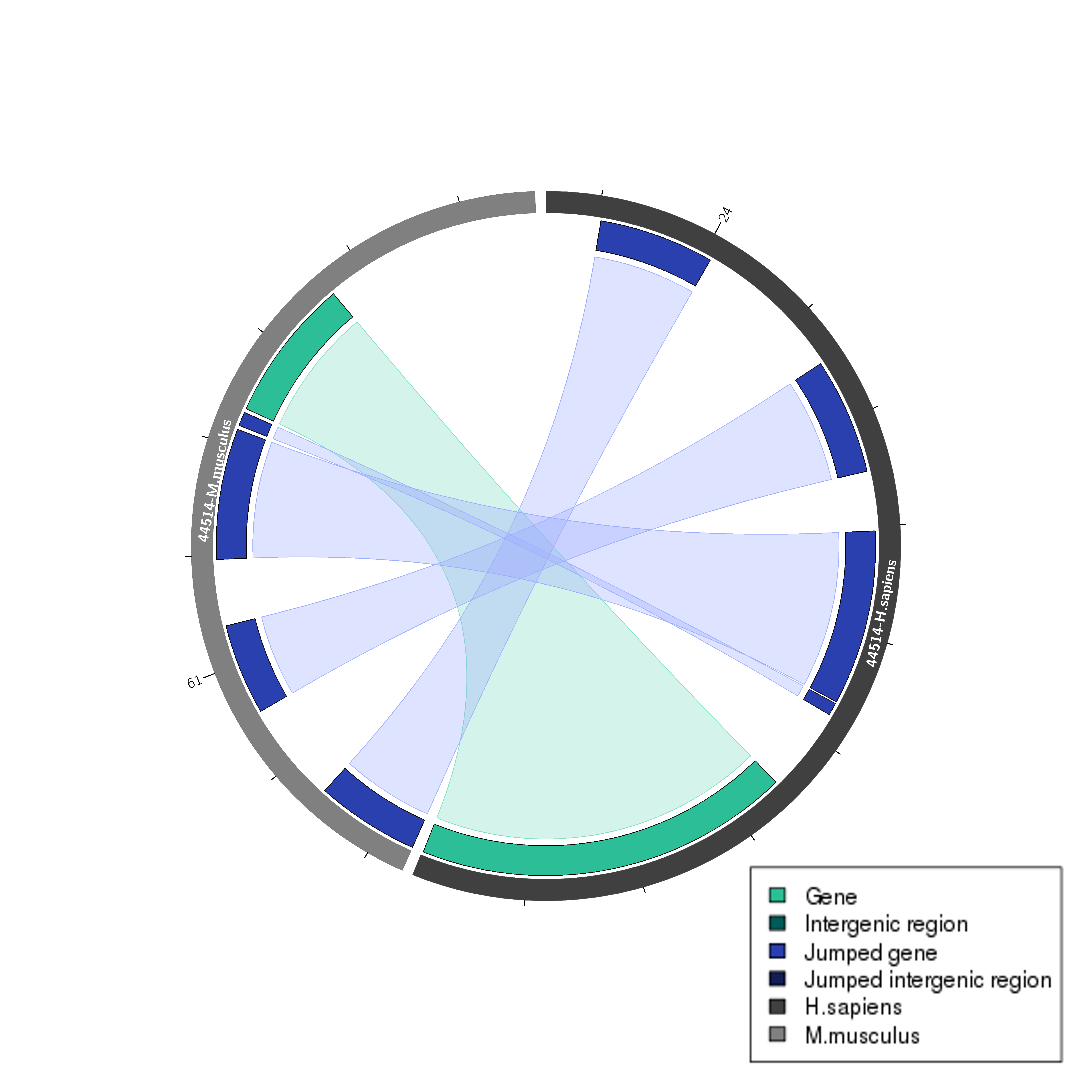

Supplement: Figure S5 — A syntenic region (ENSEMBL identifier 44514) in human (dark grey) and mouse (light grey) containing a large inversed segment, illustrated in blue and only one ortholog with the same orientation as the syntenic region (green). Ticks are placed at 100 kB distance and the numbers show the position on chromosomes 13 (human) and 14 (mouse) in mB. (PNG) [file pone.0112341.s005.png]

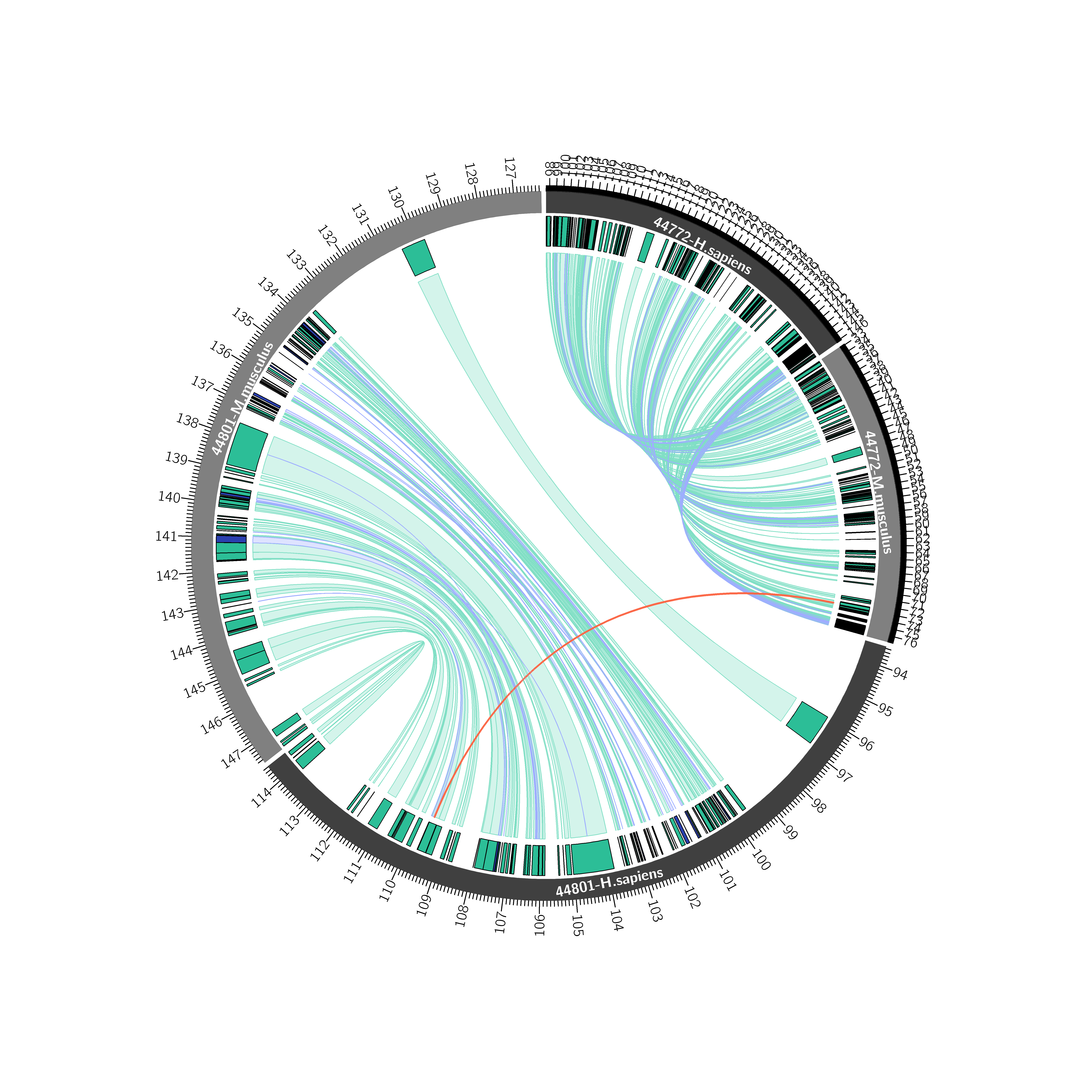

Supplement: Figure S6 — Translocation of a single gene from the human region 44801 to the mouse region 44598, shown with red line. Ticks are placed at 100 kB distance and the numbers show the positions in mB on chromosomes X in human and mouse (region 44801) as well as on chromosomes 19 in human and 7 in mouse (region 44598). (PNG) [file pone.0112341.s006.png]

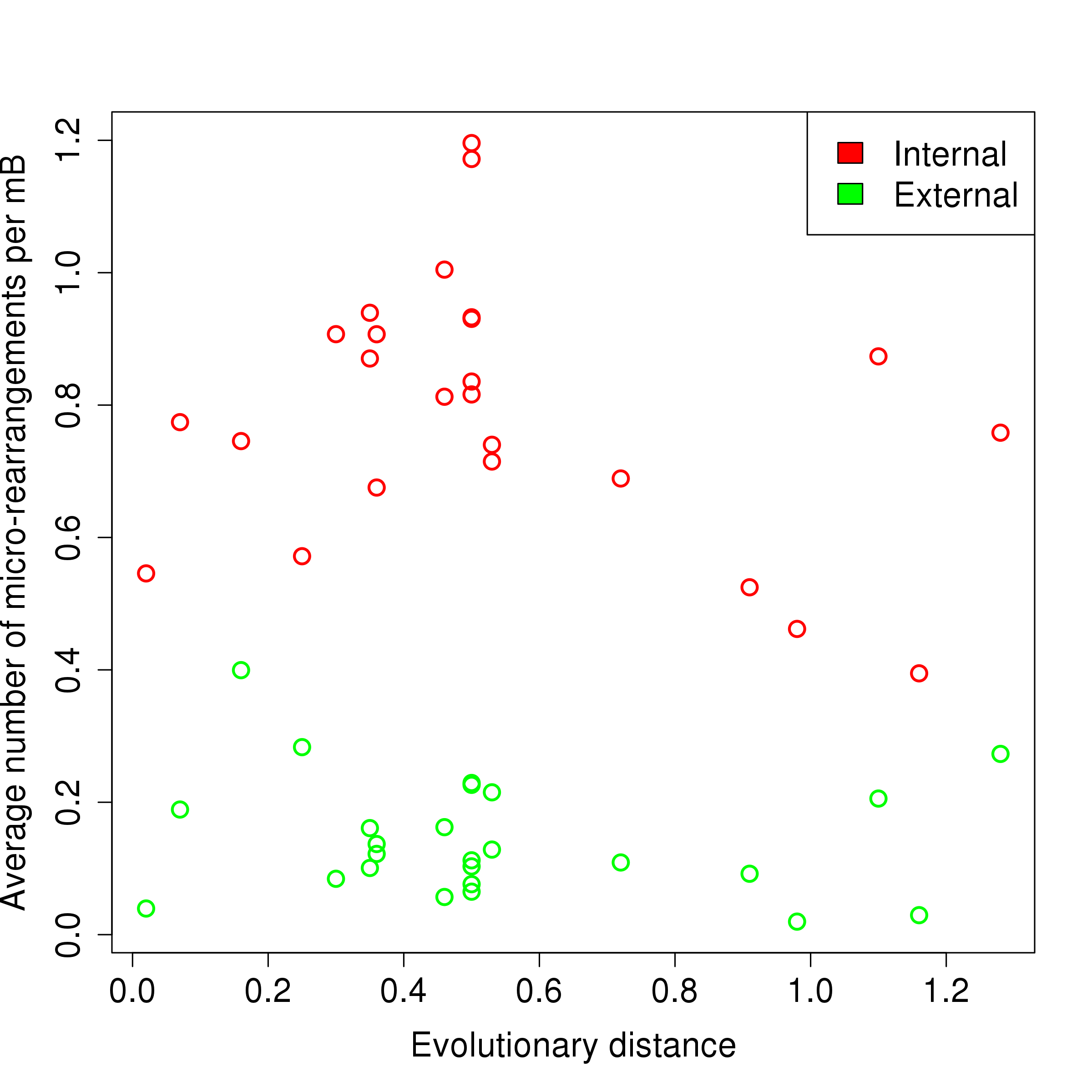

Supplement: Figure S7 — Average number of micro-rearrangements (internal and external) per megabase covered by syntenic regions versus evolutionary distance. No correlation can be observed. (PNG) [file pone.0112341.s007.png]

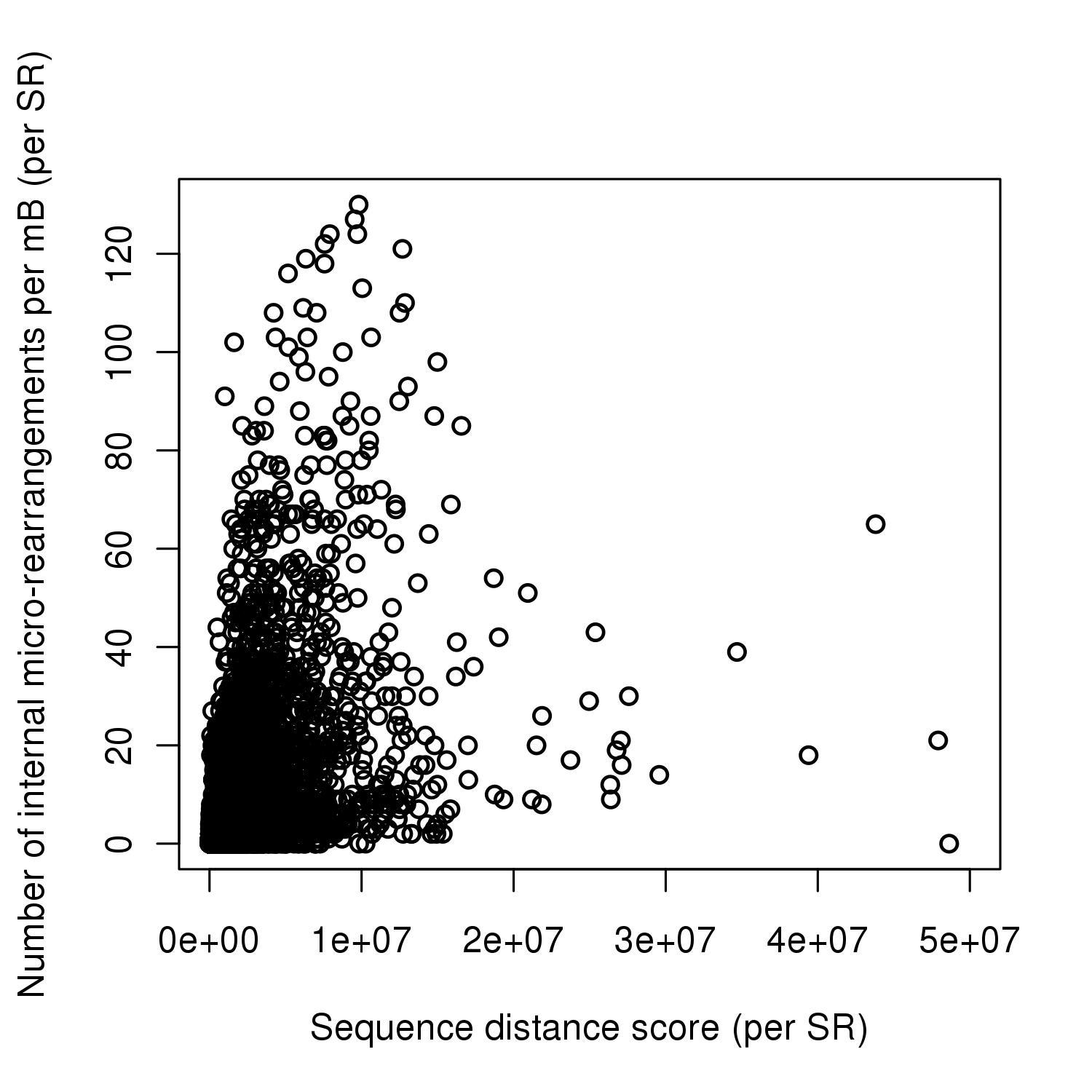

Supplement: Figure S8 — Syntenic regions (SR) with low sequence similarity (high distance score) show a trend to contain more internal micro-rearrangements per megabase (Pearson correlation coefficient 0.22). Outliers with extremely high sequence distance are not shown. (JPG) [file pone.0112341.s008.jpg]

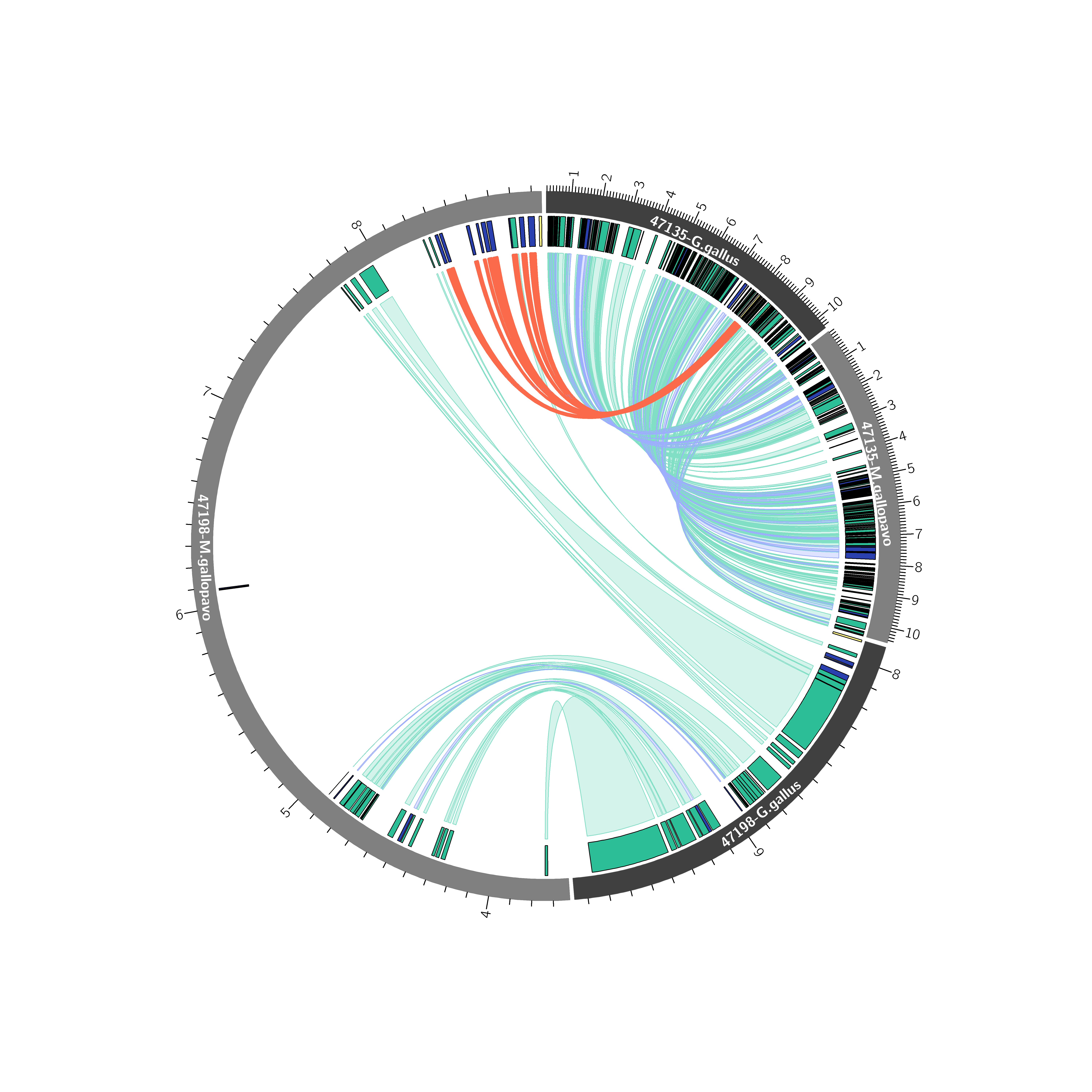

Supplement: Figure S9 — A translocation involving six consecutive genes between two syntenic regions in chicken ( G. gallus , dark grey) and wild turkey ( M. gallopavo , light grey), marked by red lines. Ticks are placed at 100 kB distance and the numbers show the positions in mB on chromosomes 6 in chicken and 8 in turkey (region 47198) as well as on chromosomes 17 in chicken and 19 in turkey (region 47135). (PNG) [file pone.0112341.s009.png]

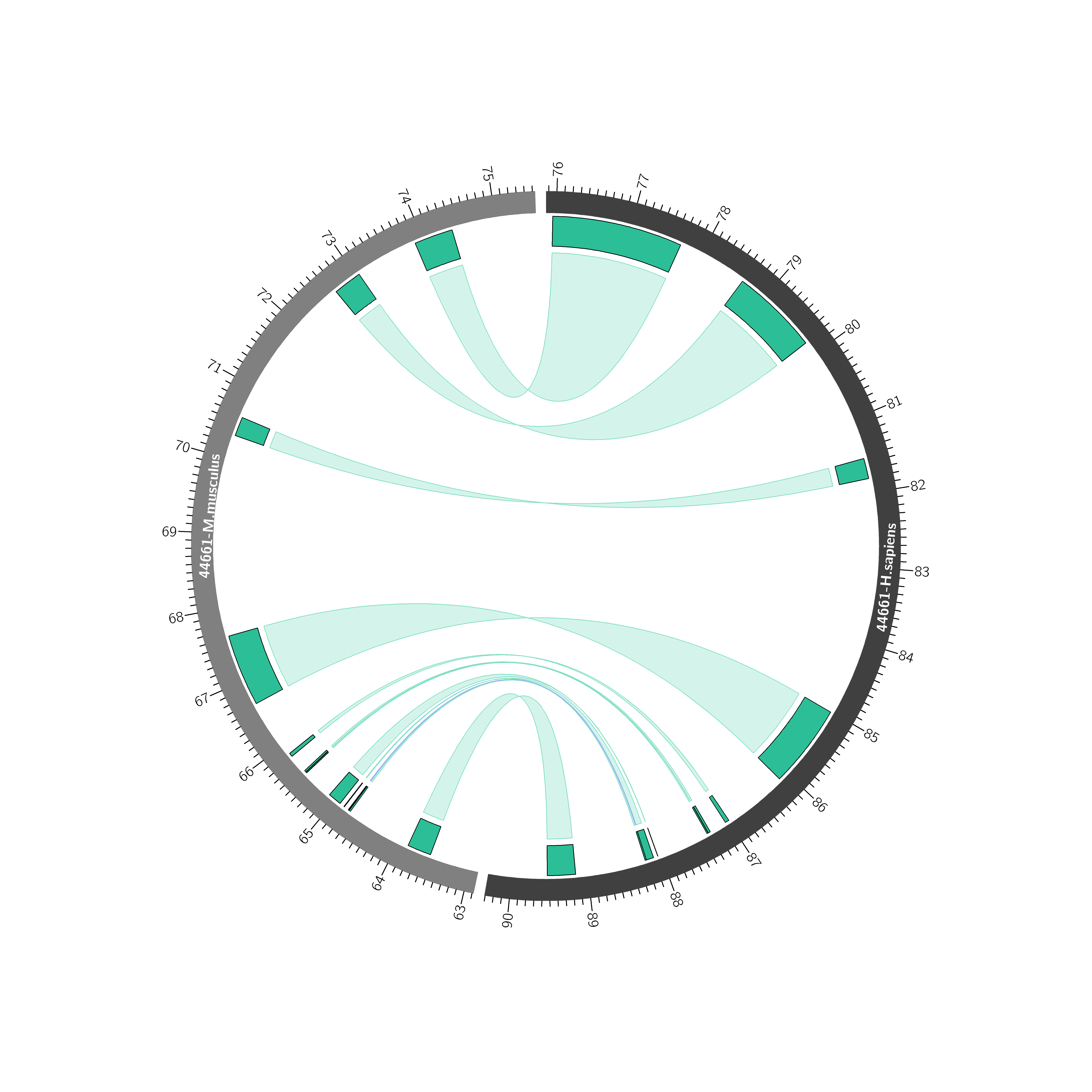

Supplement: Figure S10 — An example of an inversed syntenic region between human and mouse where CYNTENATOR detects no collinear block despite eleven genes lying in the same order in both genomes. (PNG) [file pone.0112341.s010.png]

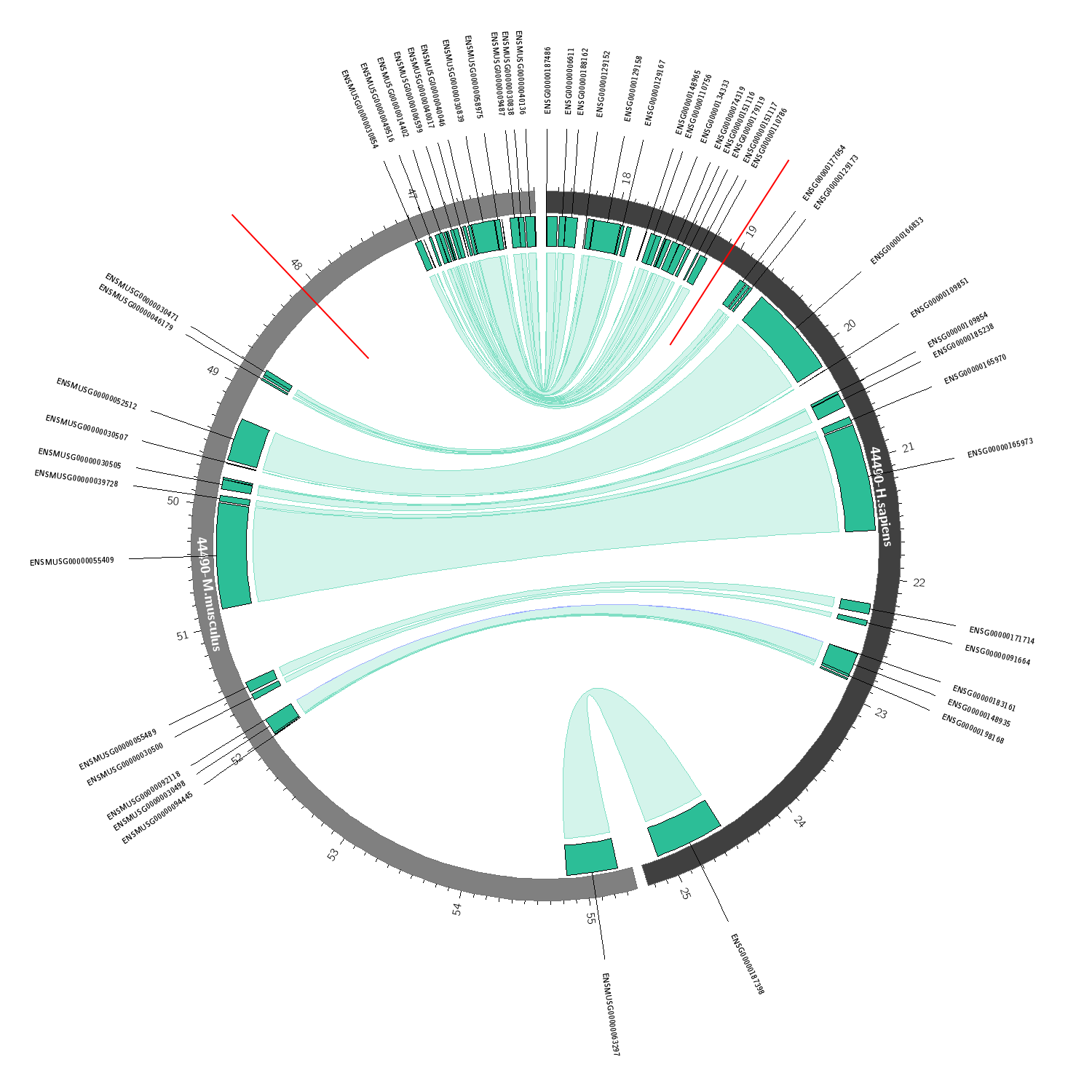

Supplement: Figure S11 — An example of a syntenic region between human and mouse where CYNTENATOR fragments the existing collinear block into two blocks (location of split marked red in both species) despite lack of genes disrupting the gene order. (PNG) [file pone.0112341.s011.png]

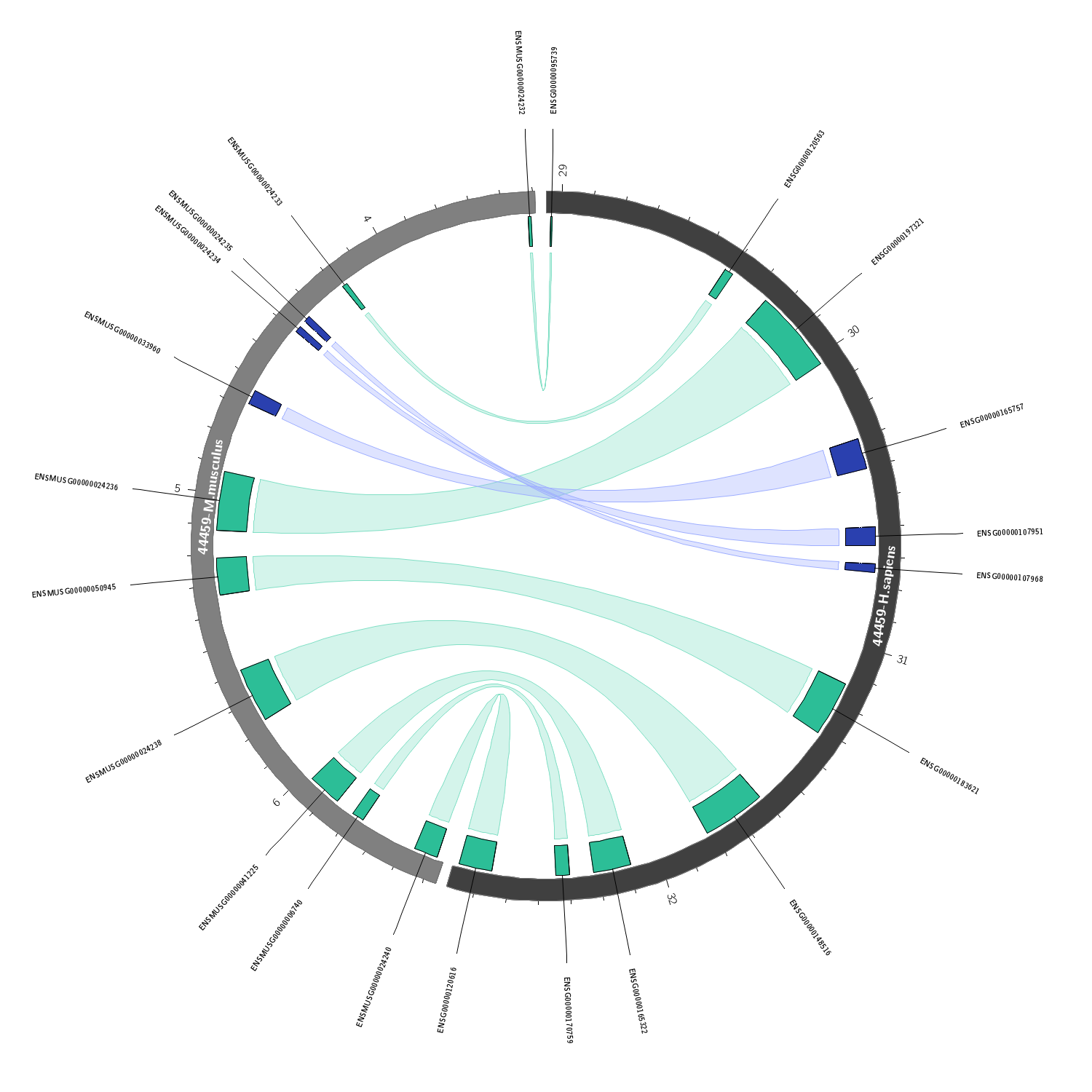

Supplement: Figure S12 — Example of a syntenic region (ENSEMBL identifier 44459) including a micro-rearrangement of three genes that is correctly identified by SyntenyMapper but ignored as a gap by i-ADHoRe. (PNG) [file pone.0112341.s012.png]
