# Supplementary material for: Analysis of Micro-Rearrangements in 25 Eukaryotic Species Pairs by SyntenyMapper
Source: PLoS One. 2014 Nov 6;9(11):e112341. doi: 10.1371/journal.pone.0112341 (PMC4223023; doi:10.1371/journal.pone.0112341)
Supplement: Table S1 — Statistics of pre-computed synteny mapping for ENSEMBL Compara (version 73). (PDF) [file pone.0112341.s013.pdf]

Table S1.

Species pair	#Syntenic regions (total)	#Orthologs (total)	#Regular syntenic regions	Avg. #genes (regular SR)	#Internal SR	Avg. #genes (internal SR)	#External SR	Avg. #genes (external SR)
Dog – Horse	2,051	16,183	201	67.02	1,458	1.59	392	1.08
Chicken – Lizard	761	5,946	261	19.12	456	1.99	44	1.16
Chicken– Wild Turkey	1,249	11,969	114	88.14	1,008	1.74	127	1.28
Human – Cow	3,181	16,723	388	32.50	2,577	1.51	216	1.03
Human – Marmoset	3,433	16,399	308	48.60	2,808	1.22	359	1.01
Human – Dog	3,145	16,399	308	38.51	2,610	1.65	227	1.03
Human – Horse	2,912	16,378	246	50.69	2,545	1.49	121	1.03
Human – Cat	2,904	16,222	265	47.63	2,499	1.38	140	1.01
Human – Chicken	2,179	11,044	423	17.91	1,565	2.07	191	1.17
Human – Gorilla	2,929	17,592	84	172.85	2,650	1.09	195	1.0
Human – Macaque	3,059	16,927	219	62.66	2,508	1.14	332	1.02
Human – Opossum	2,796	12,472	496	19.33	2,128	1.27	172	1.01
Human – Mouse	3,243	16,666	356	34.27	2,805	1.56	82	1.02
Human – Platypus	518	1,948	210	7.33	302	1.33	6	1.17
Human – Rabbit	2,107	11,529	229	37.20	1,720	1.66	158	1.02
Human – Chimp	2,673	17,265	139	94.73	2,419	1.65	115	1.02
Human – Orang-Utan	2,861	16,801	150	90.83	2,598	1.18	113	1.02
Human – Rat	3,353	16,413	546	21.60	2,540	1.71	267	1.05
Human – Pig	3,919	14,457	357	24.68	3,449	1.60	113	1.08
Mouse – Cow	2,578	16,482	439	30.89	1,941	1.40	198	1.02
Mouse – Chicken	1,979	10,94	502	15.69	1,251	2.24	226	1.11
Mouse - Dog	2,608	16,106	364	35.46	1,961	1.49	283	1.0
Mouse – Platypus	473	1,897	235	6.73	230	1.34	8	1.0
Mouse – Pig	3,524	14,115	397	22.25	2,984	1.72	143	1.04
Mouse – Rat	3,329	18,860	554	26.56	2,150	1.63	625	1.03
