# Supplementary material for: Analysis of Micro-Rearrangements in 25 Eukaryotic Species Pairs by SyntenyMapper
Source: PLoS One. 2014 Nov 6;9(11):e112341. doi: 10.1371/journal.pone.0112341 (PMC4223023; doi:10.1371/journal.pone.0112341)
Supplement: Table S2 — Evolutionary distance and genome coverage by syntenic regions for all species pairs. SR: Syntenic region. (PDF) [file pone.0112341.s014.pdf]

Table S2.

Species pair	Evolutionary distance	Percentage of longer genome covered by ENSEMBL syntenic regions
Dog – Horse	0.25	94.84%
Chicken – Lizard	0.91	53.98%
Chicken – Wild Turkey	-	94.48%
Human – Cow	0.36	88.66%
Human – Marmoset	-	89.82%
Human – Dog	0.35	89.29%
Human – Horse	0.30	89.21%
Human – Cat	0.35	89.34%
Human – Chicken	1.10	77.82%
Human – Gorilla	-	91.95%
Human – Macaque	0.07	90.77%
Human – Opossum	0.72	76.70%
Human – Mouse	0.46	87.52%
Human – Platypus	0.98	20.43%
Human – Rabbit	0.36	73.13%
Human – Chimp	0.02	85.35%
Human – Orang-Utan	-	83.73%
Human – Rat	0.46	87.11%
Human – Pig	-	87.85%
Mouse – Cow	0.53	87.50%
Mouse – Chicken	1.28	73.68%
Mouse – Dog	0.53	88.42%
Mouse – Platypus	1.16	19.91%
Mouse – Pig	-	87.08%
Mouse – Rat	0.16	93.58%
